# Manganese levels in infant formula and young child nutritional beverages in the United States and France: Comparison to breast milk and regulations

**DOI:** 10.1371/journal.pone.0223636

**Published:** 2019-11-05

**Authors:** Seth H. Frisbie, Erika J. Mitchell, Stéphane Roudeau, Florelle Domart, Asuncion Carmona, Richard Ortega

**Affiliations:** 1 Department of Chemistry and Biochemistry, Norwich University, Northfield, VT, United States of America; 2 Better Life Laboratories, Incorporated, East Calais, VT, United States of America; 3 University of Bordeaux, Centre d’Etudes Nucléaires de Bordeaux Gradignan (CENBG), Gradignan, France; 4 Centre National de la Recherche Scientifique (CNRS), Institut National de Physique Nucléaire et de Physique des Particules (IN2P3), CENBG, Gradignan, France; Università degli Studi di Milano, ITALY

## Abstract

Exposure to high levels of manganese (Mn) in children has recently been associated with adverse neurodevelopmental effects. Current infant formula regulations for Mn content were set between 1981 (United States), 2006 (European Union, France), and 2007 (Codex Alimentarius) prior to the publication of much of the growing body of research on the developmental neurotoxicity of Mn. In this study, we sought to measure the concentrations of Mn in some infant formulas and young child nutritional beverages available on the United States (US) and French markets using ion beam analysis by particle induced X-ray emission (PIXE) spectrometry and then compare the analytical results to concentrations reported in the literature for breast milk and applicable infant formula regulations and guidelines. We were particularly interested in measuring Mn concentrations in product types for which there is very little data from previous surveys, especially soy-based, rice-based, goat-milk based, chocolate-flavored, and nutritional beverages for young children that are not regulated as infant or follow-on formulas (e.g. “toddler formulas” and “toddler powders”). We purchased 44 infant formulas and young child nutritional beverage products in the US and France with varying protein sources (cow-milk, goat-milk, soy, rice) labelled for birth to 3 years. We selected these samples using maximum variation sampling to explore market extremes to facilitate comparisons to regulatory limits. Since this sampling method is non-probabilistic, other inferences cannot be made beyond this set of samples to the overall markets. We used ion beam analysis to measure the concentrations of Mn in each product. The range of measured Mn concentrations in the products is 160–2,800 μg/L, substantially higher than the 3–6 μg/L mean Mn concentration reported in human breast milk. All products satisfied national and Codex Alimentarius Commission (CAC) international standards for minimum Mn content in infant formulas; however, 7/25 of the products purchased in the US exceeded the CAC Guidance Upper Level of 100 μg Mn/kcal for infant formula.

## Introduction

Manganese (Mn) is both an essential nutrient and a toxic element. The essentiality of Mn is reflected in national and international infant formula and food policies, which stipulate minimum Mn concentrations [1–3]. While Mn toxicity due to occupational exposures has been known for almost 200 years [4], the developmental neurotoxicity of Mn has only recently begun to be explored [4–8]. Infant formula regulations have not yet been adjusted to reflect this growing body of research on the developmental neurotoxicity of Mn; while maximum Mn content is regulated for infant formulas in some jurisdictions such as the European Union and France [3,9], in other jurisdictions, such as the United States (US), there is no regulatory maximum for Mn in infant formulas [1].

Serious concerns have recently been raised about relatively high Mn exposures and possible associated adverse effects on child neurodevelopment. Children exposed to higher levels of Mn compared to other children have been found to have impaired cognitive development [4,5,7,10–21] lower IQ or intelligence scores [4,6,7,10,12,13,22–31], impaired memory function [4, 6,10,24,32–37], lower academic skills or achievement [5,6,25], impaired executive function [6,10,12,24,32], lower visual-spatial ability [32,38], impaired motor function [4–6,24,25,36,39–41], impaired olfactory function [5,6,24], atypical brain structure or function [42,43], and relatively high Mn exposures are suspected of increasing the risk of attention deficits, hyperactivity, or attention deficit hyperactivity disorder (ADHD) [5–7,12,13,24,33,36,44], and other behavior and attention problems [4,6,7,12,16,25,45–47]. Some links that have been reported between high Mn levels and certain neurodevelopmental effects are not fully conclusive. Levels of Mn exposure that constitute excess for infants or young children as demonstrated by adverse health effects have not yet been rigorously identified in the literature; however, a benchmark dose for Mn in drinking water associated with decreased IQ has recently been calculated for school-aged children [22]. Many studies [13–21,26–43,45–47] and reviews [4–7, 10–12,22–25,44] of Mn exposures in children have found significant associations between higher Mn exposures and adverse neurodevelopmental outcomes. In contrast, some studies [48–52] and one review [53] have not found significant effects. The Mn concentrations found in infant formula and young child nutritional beverages in this study have not been compared to Mn intakes associated with adverse effects on child neurodevelopment.

In infants younger than weaning age (4–6 months), breast milk or infant formula typically constitutes the sole source of nutrition. Human breast milk is considered to be the optimal food for infants, providing all necessary macro- and micro-nutrients in sufficient quantities to sustain health and development through the age of weaning [54]. Breast milk usually contains 2–6 μg/L of Mn [55]. Infant formula has been reported to contain 195 times more Mn than the levels usually found in breast milk [56–58].

There are very few data available on Mn bioavailability in infants. In a study of 2–16 week-old infants, the retention of Mn was higher for formula-fed infants than for breast-fed infants due to the higher Mn content of infant formula [59]. It is noteworthy that in laboratory studies on Mn content in infant formula, high purity water with very low Mn content is used to reconstitute the infant formula. However, if infant formula is reconstituted with tap water, Mn from the tap water may further increase the total Mn content of the formula and the total Mn intake for the infant [56]. Moreover, Mn absorption rates are higher in neonates, 16–37% [59], compared to roughly 3% in adults [7]. Infants and especially neonates are further susceptible to Mn toxicity due to transiently diminished biliary excretion, which is the major route of Mn excretion in humans [7]. Overall, these data suggest that Mn intake and retention in children fed with infant formula are much higher than in children fed with breast milk or in adults.

In this study, we sought to measure the concentrations of Mn in infant formulas and young child nutritional beverages available on the US and French markets using ion beam analysis by particle induced X-ray emission (PIXE) spectrometry and then compare the analytical results to concentrations reported in the literature for breast milk and applicable child feeding regulations and guidelines. Because of their wide availability in analytical chemistry laboratories, especially in industry, ICP-AES (inductively coupled plasma-atomic emission spectrometry) and FAAS (flame atomic absorption spectrometry) are the official methods for Mn analysis in infant formula [60,61]. Other analytical methods can be used as well, among them PIXE analysis [62–69], a less widespread technique that requires a particle accelerator. PIXE is a multi-elemental analytical technique that can be performed to quantify trace element content in a large variety of samples, including food samples, with minimum sample processing and high analytical accuracy.

Previous surveys of Mn concentrations in infant formulas often report higher Mn content in soy-based formulas than milk-based samples [56,58,70] but with few soy-based samples tested. In the present study, we were particularly interested in measuring Mn concentrations in product types for which there is very little data from previous surveys, especially soy-based, rice-based, goat-milk based, chocolate-flavored, and nutritional beverages for young children that are not regulated as infant or follow-on formulas (e.g. “toddler formulas” and “toddler powders”). Our hypothesis was that the Mn concentrations would be within the limits of infant formula regulations but might differ from the reported ranges in breast milk and that the protein source of the products might affect Mn content. We did not test the link between high Mn intake in children and brain disorder and adverse neurodevelopmental effects.

## Material and methods

### Selection of samples

A total of 25 products were purchased in the United States (US). Seventeen of these 25 products were labelled for use by infants (ages 0–12 months) [1] and 8 of these 25 products were labelled for use by young children (ages 1 year and older). Fifteen of the 17 infant products were standard infant formulas, and 2 of the 17 products were special medicinal “exempt infant formulas” [1]. Of the 8 products labelled for use by young children, 5 were “toddler” products, 2 were medical complete nutritional beverages, and 1 was a follow-on formula [2]. Of the 25 products obtained from the US market, 13 were cow milk-based, 5 were soy protein-based, 3 were goat-milk based, 3 were amino acid-based, and 1 was rice-based.

A total of 19 products were purchased in France. Seventeen of these 19 products were standard infant or follow-on formulas and 2 of these 19 products were liquid infant complementary foods [3,9]. The 2 liquid complementary foods from France were selected for this study in order to include samples containing chocolate or rice from the French market. In general, it was much more difficult to find formulas containing chocolate or rice in the French market than in the US market. We were not able to locate any powdered formula products in the French market that were chocolate flavored or rice-based. We were not able to obtain a soy-based or amino acid-based powdered formula in France.

For the purpose of this paper, all products labeled for use by young children 1 year and older, including follow-on formulas, medical complete nutritional beverages, liquid complementary foods, and “toddler” formulas, powders, or beverages are termed “young child nutritional beverage products”.

All 44 of these samples were purchased in the US and France using maximum variation sampling [71]. More specifically, these samples were purposively selected to yield a wide range of Mn concentrations. That is, these samples were selected to contain supplemental Mn, soy, rice, cow milk, goat milk, and chocolate, and to cover a wide range of ages, from birth to 3 years. This sampling method was designed to explore market extremes as well as apparently typical products in order to facilitate comparisons to regulatory compliance. Study goals were to determine the high and low ranges of Mn content in formula products rather than overall market trends. Since the sampling method is non-probabilistic, other inferences cannot be made beyond this set of samples to the overall market [71]. This sampling method was not designed to evaluate the batch to batch variability of any individual product.

### Preparation of samples

Forty-two samples were powdered products and 2 samples were solutions. Solutions and suspensions (of the partially soluble products) of all 42 powdered samples were prepared for analysis using laboratory-grade plasticware. One solution or suspension was made for each sample of powdered product. A step-by-step protocol of infant formula sample preparation for PIXE/RBS determination of element concentrations has been deposited in the protocol.io repository [dx.doi.org/10.17504/protocols.io.5qxg5xn]. More specifically, between 1.0000 grams (g) and 1.0099 g of powdered product, and 10.00 milliliters (mL) of ultra-trace elemental analysis grade water (Fisher Chemical, Catalog No. W9-500) were delivered to a centrifuge tube, mixed, and stored at 4°Celsius (C) until spotted on polycarbonate sample holders for ion beam analysis by PIXE spectrometry. The PIXE method can be applied to quantify trace elements in food samples [62]. PIXE has also been used to study trace element content in human breast milk and infant formulas [63–69].

Each of the 42 sample solutions or suspensions and each of the 2 liquid samples was mixed and spotted in 3 different locations on a single sample holder. Each spot was delivered with a 1.0 microliter (μL) air displacement pipet. These spots were dried at room temperature in a laminar flow hood. This process was repeated until a total of 3 spots were superimposed on each of the 3 different locations of each sample holder.

### PIXE/RBS analysis

Each of the 3 spots on the sample holders was analyzed by PIXE; the 3 PIXE measurements were averaged to give a single reported concentration for each sample. These PIXE analyses were performed at the *Centre d’Etudes Nucléaires de Bordeaux Gradignan* (CENBG; Nuclear Studies Center of Bordeaux Gradignan) using the high-resolution beamline at the *Applications Interdisciplinaires de Faisceaux d’Ions en Région Aquitaine* (AIFIRA; Interdisciplinary Applications of Ion Beams in the Aquitaine Region) facility [72]. A Singletron particle accelerator system (High Voltage Engineering Europa B.V.) delivered a 3.0 megaelectron volt (MeV) proton (H^+^) beam at approximately 300 picoamperes (pA) to the dried sample.

The principle of PIXE analysis as pioneered by Johansson and Johansson [73] is based on the interaction of accelerated charged particles, usually H^+^, with the sample. In brief, the H^+^ beam can ionize inner shell electrons from the atoms in the sample. For a specific element, an x-ray with a characteristic energy is emitted when an electron from an outer shell replaces an ionized electron from an inner shell. For elements heavier than sodium (Na), these x-rays were measured and averaged by 2 identical lithium-drifted silicon x-ray detectors, Si(Li). Each of these 2 detectors was at a 45° angle to the H^+^ beam; that is, the H^+^ beam bisects a 90° angle between the 2 detectors and the sample at the vertex [74]. Each detector was 2 cm from the sample. A 100 μm thick carbon foil funny filter, a filter with a pinhole drilled at its center, was placed between the sample and each of the 2 x-ray detectors. This attenuates the signal of the more abundant light elements and decreases the detector’s dead time. Both Si(Li) x-ray detectors had a 145 electron volt (eV) energy resolution at 5.92 kiloelectron volt (keV), the Mn KL2,3 x-ray emission line. This energy resolution enables the clear identification of the Mn KL2,3 x-ray emission line for the accurate quantification of Mn. For quantitative analysis of PIXE data, the system is calibrated with a set of thin film standards containing certified concentrations of reference elements (Micromatter^™^). Micromatter^™^ standards consist of the certified elements, each with an areal density of about 50 μg/cm^2^, deposited on a 6 mm Mylar foil. PIXE allows the determination of element concentration expressed in terms of the areal mass of the element (μg/cm^2^).

In order to quantify the element concentration in terms of μg of the element per g of sample, a second ion beam analysis technique, Rutherford backscattering spectrometry (RBS), is carried out simultaneously to PIXE [74,75]. For the RBS analysis, a fraction of the protons from the H^+^ beam is repelled by the positively charged atomic nuclei in the sample [76]. The energies of the backscattered protons were measured with a 25 mm^2^ silicon passivated detector, 12 keV full width at half maximum at 5.486 MeV using the 241 isotope of americium (^241^Am). This detector was at a 45° angle to the H^+^ beam; that is, the H^+^ beam forms a 45° angle to the detector with the sample at the vertex [74]. This detector was 2 cm from the sample. RBS measures the atomic concentrations of carbon (C), nitrogen (N), and oxygen (O), the main components in biological samples that cannot be measured by PIXE, which is sensitive to elements of atomic number > 11. Therefore, for sample matrixes, such as milk, which are composed mainly of light elements, RBS allows the determination of the total areal mass of the sample expressed in g/cm^2^. The combination of PIXE and RBS allows the quantification of element concentrations in μg/g of dry mass.

In addition, RBS measures the number of H^+^ that interact with the sample during the analysis; this parameter is required to quantify the concentrations of the elements that are detected by PIXE [74]. Therefore, the simultaneous analysis of PIXE and RBS enables the quantification of the concentrations of elements in solid samples [74,75,77]. GupixWin software [78] and SIMNRA software [79] were used to calculate these concentrations.

The accuracy of PIXE/RBS quantitative analysis was assessed with the analysis of a National Institute of Standards & Technology (NIST) Standard Reference Material® (SRM), 1849a Infant/Adult Nutritional Formula. This assessment was used to validate the entire analytical procedure for Mn quantification, from sample preparation through data treatment.

The measured concentrations were converted to μg of element per liter (L) of prepared product following the instructions from the manufacturer’s labels. This was done using the mass of solid product measured 3 times to the nearest 0.0001 g with an analytical balance, and the final volume of the prepared formula measured 3 times to the nearest 0.1 mL with a graduated cylinder. For formula sold in the US, the legal definition of 1 fluid ounce equals 30 mL was used for this preparation since product labels must use this definition when reporting element concentrations [80].

Finally, the measured concentrations were converted to μg of element per 100 kilocalories (kcal) of prepared product. This was done using the reported kcal/mass of dried product or final volume of prepared product from the manufacturer’s label.

## Results and discussion

### The analysis of Standard Reference Material® by particle induced x-ray emission, Rutherford backscattering spectrometry

National Institute of Standards & Technology (NIST) Standard Reference Material® (SRM) 1849a, Infant/Adult Nutritional Formula is the current industry standard for testing infant formula in the United States [81]. In this study, NIST SRM 1849a Infant/Adult Nutritional Formula was analyzed 3 times to assess the accuracy of PIXE/RBS for measuring the concentrations of representative elements in infant formulas and young child nutritional beverage products. NIST SRM 1849a was processed in exactly the same manner as the samples of infant formula and young child nutritional beverages in this study. Relative error was used to assess this accuracy (see Eq 1) [82].

$$Relative\;Error=E_{r}=\frac{\left(\bar{x}-x_{t}\right)}{x_{t}}\times 100\%$$ (1)

The certified mass fraction value for manganese in NIST SRM 1849a is 49.59 ± 0.97 μg/g. The mean and sample standard deviation for the 3 analyses of Mn by PIXE are 49.48 ± 1.61 μg/g, respectively. In this case, $\bar{x}$ is the sample mean for Mn in μg/g, and *x_t_* is the true or certified mass fraction for Mn in μg/g. The relative error for Mn was -0.22%. This -0.22% relative error and 1.61 μg/g sample standard deviation for the NIST SRM are estimates of the accuracy and precision for the measurement of Mn in each container of sample in this study, respectively.

NIST SRM 1849a contains more Mn than the infant products in this study; the certified mass fraction is 49.59 ± 0.97 μg/g for SRM 1849a, and mass fractions ranged from 1.3 μg/g to 32 μg/g for all the samples in this study. This might be a result of SRM 1849a Infant/Adult Nutritional Formula being designed by NIST to test both infant and adult nutritional formula, instead of just infant formula. Regardless, SRM 1849a is the current industry standard for testing infant formula in the United States [81]. Therefore, we used the same standard of comparison as industry laboratories use for product labeling under the Infant Formula Act of 1980.

### The concentrations of manganese in infant formulas and young child nutritional beverage products from the US and French markets

The measured concentrations of Mn in each of the 25 samples from the US market are shown in Table 1, along with the minimum, maximum, number of samples, the protein source, the labeled age range of the product, and whether the product was labeled for special medical purposes or contained chocolate. These parameters for the 19 samples from the French market are shown in Table 2.

**Table 1:** Mn content by PIXE/RBS spectrometry for the samples from the US market.

ID	Mn (μg/g)	Mn (μg/L)	Mn (μg/100 kcal)	Supplemental Mn	Protein Source	Chocolate	Labeled Age Range	Medical
**US01**	1.8	230	36	No	Cow^a^	No	infant	No
**US02**	2.7	400	55	No	Cow^a^	No	6 months +	No
**US03**	2.3	310	47	Yes	Cow^a^	No	0–12 months	No
**US04**	1.3	160	26	No	Cow	No	toddler	No
**US05**	7.9	1,000	170	No	Soy	No	toddler	No
**US06**	3.4	430	65	Yes	Cow	No	0–12 months	No
**US07**	2.7	320	48	Yes	Cow^a^	No	infant	No
**US08**	7.4	1,600	160	Yes	Amino acids	No	1+ years	Yes
**US09**	2.6	340	50	Yes	Goat	No	1–3 years	No
**US10**	11	2,100	240	Yes	Cow	Yes	1–13 years	Yes
**US11**	9.2	1,100	220	No	Goat	Yes	13 months-8 years	No
**US12**	32	2,800	860	Yes	Rice	No	1–4 years	No
**US13**	2.2	330	47	Yes	Goat	No	1–2 years	No
**US14**	5.7	830	120	Yes	Amino acids	No	infant	No
**US15**	4.2	540	81	No	Soy	No	infant	No
**US16**	3.9	480	72	No	Soy	No	infant	No
**US17**	2.9	420	64	No	Soy	No	0–12 months	No
**US18**	1.6	210	31	Yes	Amino acids	No	0–12 months	No
**US19**	6.3	790	120	Yes	Soy	No	0–12 months	No
**US20**	1.6	230	34	Yes	Cow	No	infant	No
**US21**	2.7	340	51	Yes	Cow	No	infant	No
**US22**	2.9	420	63	Yes	Cow	No	infant	No
**US23**	2.1	260	39	Yes	Cow	No	infant	No
**US24**	2.5	320	48	Yes	Cow	No	infant	No
**US25**	2.1	260	39	Yes	Cow	No	infant	No
**Min**	1.3	160	26					
**Max**	32	2,800	860					
**Mean^b^**	**5.0**^b^	**650**^b^	**110**^b^					
**Median^b^**	**2.7**^b^	**400**^b^	**55**^b^					
***s*^b^**	**6.2**^b^	**650**^b^	**170**^b^					

a Contains rice

b Applies to this dataset only. Since the sampling method was non-probabilistic, inferences to the overall market are not intended and should not be made.

**Table 2:** Mn content by PIXE/RBS for the samples from the French market.

ID	Mn (μg/g)	Mn (μg/L)	Mn (μg/100 kcal)	Supplemental Mn	Protein Source	Chocolate	Labeled Age Range	Medical
FR01	1.7	240	35	Yes	Cow	No	0–6 months	No
FR02	2.1	290	46	Yes	Cow	No	12+ months	No
FR03	1.7	230	37	Yes	Cow	No	6–12 months	No
FR04	2.3	290	46	Yes	Goat	No	6+ months	No
FR05	1.7	220	34	Yes	Goat	No	1+ years	No
FR06	2.4	310	46	Yes	Cow	No	0–6 months	No
FR07	2.7	350	53	Yes	Cow	No	0–6 months	No
FR08	5.9	1,200	140	No	Cow	Yes	6+ months	No
FR09	4.6	860	100	No	Cow^a^	No	6+ months	No
FR10	2.5	350	53	Yes	Goat	No	6+ months	No
FR11	2.0	320	42	Yes	Cow	No	6–12 months	No
FR12	2.6	390	55	Yes	Cow	No	10+ months	No
FR13	1.5	200	32	Yes	Cow	No	1–3 years	No
FR14	2.1	300	44	Yes	Cow	No	6–12 months	No
FR15	2.0	280	40	Yes	Cow^a^	No	6 months +	No
FR16	2.0	320	47	Yes	Cow	No	6–12 months	No
FR17	1.9	290	44	Yes	Cow	No	1+ years	No
FR18	2.5	340	52	Yes	Cow	No	12+ months	No
FR19	4.4	560	93	Yes	Cow^a^	No	0–36 months	No
Min	1.5	200	32					
Max	5.9	1,200	140					
**Mean**^b^	**2.6**^b^	**390**^b^	**55**^b^					
**Median**^b^	**2.1**^b^	**310**^b^	**46**^b^					
***s***^b^	**1.1**^b^	**250**^b^	**27**^b^					

a Contains rice

b Applies to this dataset only. Since the sampling method was non-probabilistic, inferences to the overall market are not intended and should not be made.

The concentrations in Tables 1 and 2 are given in 3 equivalent units, μg of element per g of dried product (μg/g), μg of element per L of prepared product (μg/L), and μg of element per 100 kcal of prepared product (μg/100 kcal). In prior surveys of infant formulas and cow, goat, and human milks, a vast array of units, such as μg/quart [83] and nanomoles per L [84] have been used, requiring unit conversions in order to compare results across studies. In this study, results are reported in μg/g because these units are directly measured by PIXE/RBS and many other instrumental methods.

In this study, results are also reported in μg/L because these units are readily comparable to many prior surveys of liquid milks [85–87]. The μg/L were calculated from the measured μg/g by PIXE/RBS and the measured mass of dried product that was used to make a measured final volume of prepared product according to the instructions on the product labels. Each measurement was done 3 times and averaged; each average was used to convert μg/g to μg/L.

In this study, results are also reported in μg/100 kcal because these units are used in policies and regulations [1,2,88–91]. The regulations refer to energy units (kcals) so that they automatically scale to an infant’s weight and total energy requirements. Units given by kcals can be readily used to tabulate total intake of nutrients in both single-item and mixed diets that include both solid and liquid foods. The μg/100 kcal were calculated from the measured μg/g by PIXE/RBS and the reported kcal/mass of dried product or final volume of prepared product from the manufacturer’s label.

### Ingredients and the concentrations of manganese in infant formulas and young child nutritional beverage products

The total Mn concentration of an infant formula or young child nutritional beverage product includes any supplemental Mn, if added by the manufacturer, and the Mn from all of the other ingredients. Manganese is often added by the manufacturer to ensure that the Mn concentration of the final product is greater than the minimum required Mn concentration set by regulations. If a product is supplemented with Mn, then it is impossible to assign the total Mn concentration of the final product to any given ingredient, such as soy, rice, cow milk, goat milk, or chocolate. Therefore, all products with supplemental Mn in our survey were classified as “supplemented” for the purpose of assessing the effect of major ingredients on the total Mn concentration of each product. Products without supplemental Mn that contained soy, rice, cow milk, or chocolate were classified as “soy”, “rice”, “cow milk”, or “chocolate”, respectively.

**Fig 1:**
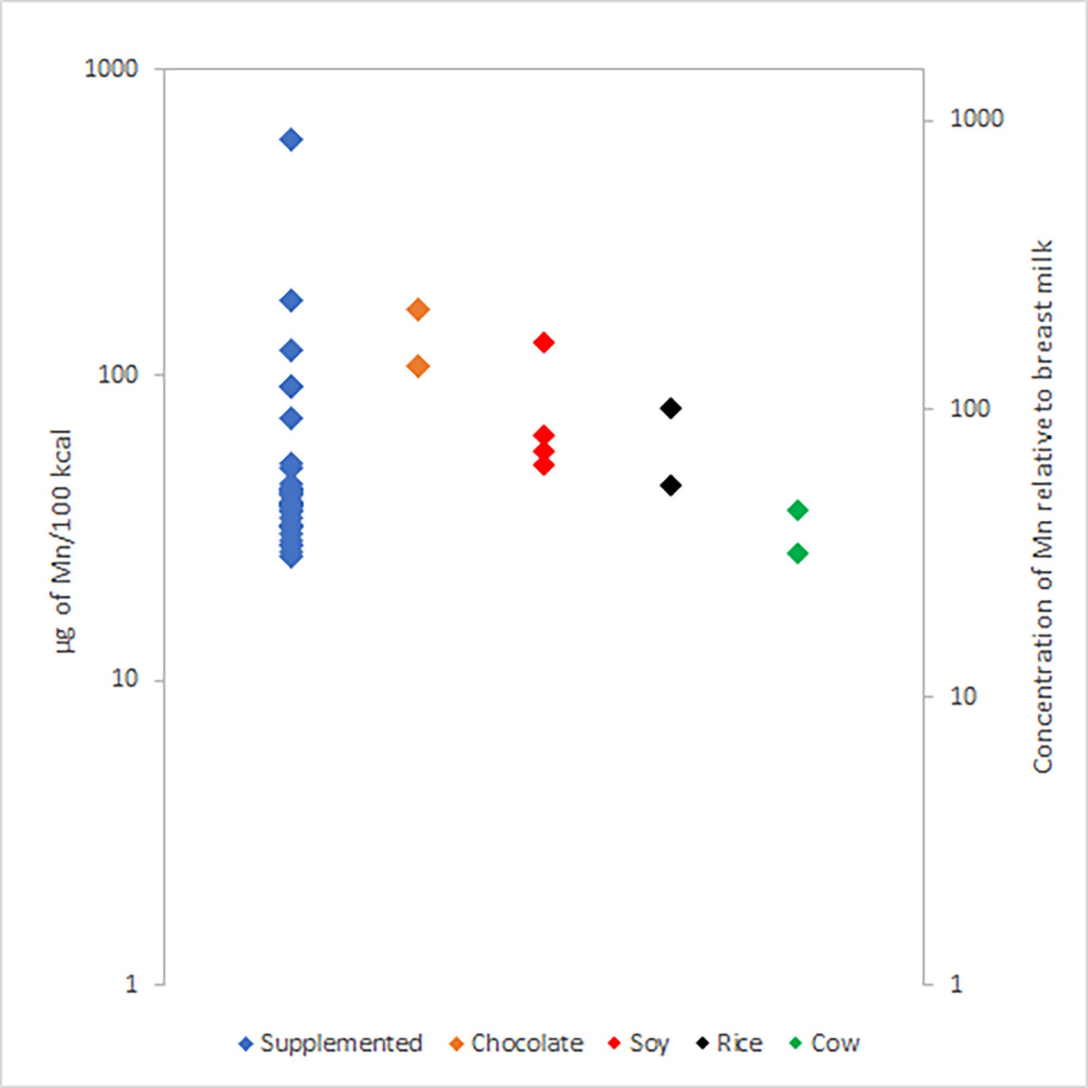
The minimum and maximum concentrations of Mn of prepared infant formulas and young child nutritional beverage products according to ingredient classes. These concentrations are also given relative to the concentration of Mn in breast milk [92].

In the 44 samples that we tested, 4 were soy-protein based without supplemental Mn, 2 contained rice without supplemental Mn, 2 were cow milk-protein based without supplemental Mn, and 2 contained chocolate without supplemental Mn; the remaining 34 samples contained supplemental Mn (see Fig 1). The minimum, maximum, and number of samples (*n*) for supplemented and soy products are shown in Table 3; cow, rice and chocolate products are not included in Table 3 due to the low number of samples in these categories.

**Table 3:** Ranges of Mn in samples according to ingredients. Samples with supplemental Mn.

Min	1.5 μg/g	200 μg/L	31 μg/100 kcal
Max	32 μg/g	2,800 μg/L	860 μg/100 kcal
$\bar{x}$^a^	3.8 μg/g^a^	510 μg/L^a^	84 μg/100 kcal^a^
Median^a^	2.4 μg/g^a^	320 μg/L^a^	47 μg/100 kcal^a^
*s*^a^	5.3 μg/g^a^	570 μg/L^a^	140 μg/100 kcal^a^
N	34 samples	34 samples	34 samples
**Samples with soy protein and without supplemental Mn**			
Min	2.9 μg/g	420 μg/L	64 μg/100 kcal
Max	7.9 μg/g	1,000 μg/L	170 μg/100 kcal
$\bar{x}$^a^	4.7 μg/g^a^	620 μg/L^a^	96 μg/100 kcal^a^
Median^a^	4.1 μg/g^a^	510 μg/L^a^	77 μg/100 kcal^a^
*s*^a^	2.2 μg/g^a^	280 μg/L^a^	47 μg/100 kcal^a^
N	4 samples	4 samples	4 samples

a Applies to this dataset only. Since the sampling method was non-probabilistic, inferences to the overall market are not intended and should not be made.

In this study, 34 of the 44 products listed supplemental Mn on the label (Fig 1 and Table 3); we classified these products as “supplemented”. The addition of supplemental Mn by manufacturers is a possible reason why the largest maximum concentration of Mn in this study, 860 μg/100 kcal, is from a product that was supplemented with Mn (Fig 1 and Table 3). For comparison, a Mn concentration of 860 μg/100 kcal is about 1,000 times greater than that of breast milk, approximately 0.83 μg/100 kcal [92].

Four of the products in this study were not supplemented with Mn and used soy as a major ingredient (Fig 1 and Table 3). Soy has a relatively high native concentration of Mn [93]. Prior surveys have consistently shown that soy protein-based formulas have higher Mn concentrations than milk-based formulas [83,86,94–97]. Therefore, soy is a possible source of the relatively high maximum concentration of Mn in the 4 “soy” products, 170 μg/100 kcal (Fig 1 and Table 3). For comparison, a Mn concentration of 170 μg/100 kcal is about 200 times greater than that of breast milk [92].

Two of the products in this study were not supplemented with Mn and used rice as an ingredient (Fig 1). Rice has a relatively high native concentration of Mn [98,99]. Rice is a possible source of the relatively high concentration of Mn in these “rice” products, with a maximum concentration of 100 μg/100 kcal. For comparison, a Mn concentration of 100 μg/100 kcal is about 120 times greater than that of breast milk [92].

Two of the products in this study were not supplemented with Mn and used cow milk as an ingredient (Fig 1). Cow milk generally has a much lower native concentration of Mn than soy, rice, or chocolate, but a higher concentration of Mn than human breast milk [55,94,95, 100–107]. This is a possible reason why “cow milk” products had the lowest maximum concentration of Mn when compared to “soy”, “rice”, and “chocolate” products, 36 μg/100 kcal (Fig 1 and Table 3). For comparison, a Mn concentration of 36 μg/100 kcal is about 43 times greater than that of breast milk [92]. Similarly, the 2 “cow milk” products also had the lowest minimum concentration of Mn in this study, 26 μg/100 kcal (Fig 1 and Table 3). A Mn concentration of 26 μg/100 kcal is about 32 times greater than that of breast milk [92].

Two of the products in this study were not supplemented with Mn and used chocolate as an ingredient (Fig 1). Chocolate has a relatively high native concentration of Mn [98,104–107]. Thus, chocolate is a possible source of the relatively high maximum concentration of Mn in the 2 “chocolate” products, 220 μg/100 kcal. For comparison, a Mn concentration of 220 μg/100 kcal is about 260 times greater than that of breast milk [92].

In this study, one of the products based on goat milk did not have supplemental Mn; however, it also contained chocolate, so the effect of goat milk on total Mn concentration could not be assessed from this set of samples. Prior surveys suggest that the Mn concentration of goat milk is comparable to that of cow milk [100,108–110]; similarly, the Mn concentration of goat-milk based formulas has been shown to be comparable to that of cow-milk based formulas [96].

### Targeted ages of consumption and the concentration of manganese in infant formulas and young child nutritional beverage products

The products that we tested were labeled for use by different ages. Products sold in the French market were all labeled with a number (1, 2, 3, or 4) to indicate the intended “age stage” of the product. Stage 1 products were labeled for ages 0–6 months (or up to 36 months for special medical formulas), stage 2 for ages 6–12 months (or older), and stages 3 and 4 for ages 1–3 years (or older). In this study, 4 samples were labelled for use by infants ages 0–6 months (stage 1), 9 were labelled for use by infants ages 6 months and older (stage 2), and 6 were labelled for use by young children ages 1 year and older (stages 3 or 4). According to French law [3], the term *préparation pour nourrissons* (infant formula) applies to products intended for the first months of life to satisfy all nutritional needs of a *nourrisson* [3] (infant, a child under 12 months old). In contrast, the term *préparation de suite* (follow-on formula), is intended to be the primary liquid nutrition for a *nourrisson* (infant) who is beginning to include complementary foods in the diet. Thus, products labeled stage 1 would be classified as *préparations pour nourrissons*, products labeled stage 2 would be classified as *préparations de suite*. Products labeled stages 3 or 4 are for *enfants en bas âge* (young children, ages 1–3 years), and are also regulated by the French law regarding *préparations pour nourrissons et aux préparations de suite* (infant and follow-on formulas) [3]. The 2 liquid complementary foods that we tested from France were labeled for use by ages 6 months and older but are classified as foods, not infant or follow-on formulas according to French law [3].

Products that we tested from the US market were labeled “infant formula”, “toddler formula”, or “toddler powder”. Some infant formula products purchased in the US that were produced outside the US also bore age stage numbers, similar to the products from France, in addition to the term “infant formula”. In this study, 16 samples were labelled for use by infants ages 0 months and older, 1 was labelled for use by infants ages 6 months and older, and 8 were labelled for use by young children ages 1 year and older. In the US market, a product can only use the term “infant formula” on the label if it meets special Food and Drug Administration (FDA) requirements for infant formulas [111]. By definition, an infant formula must be a “simulation of human milk” or “a complete or partial substitute for human milk” [111]. The FDA does not distinguish between “infant formulas” and “follow-on” formulas. However, the FDA exempts certain infant formulas intended for special medical purposes (e.g. severe allergies) from standard infant formula regulations concerning nutrient contents [111]. By definition, “infants” are persons not more than 12 months old [112]. Notably, there is no legal definition of “toddler formula” or “toddler powder” in the US [112,113]. Products labeled “toddler formula” or “toddler powder” are presumably for children older than 1 year. They are covered by the regulations that apply to ordinary foods but are not subject to the special regulations for products labeled “infant formula” [112–114].

The minimum and maximum Mn concentrations for the 30 tested products intended for children less than 1 year old (products labeled stage 1 or 2 in France or “infant formula” in the US) were 1.56 μg/g and 6.32 μg/g, respectively. The minimum and maximum Mn concentrations for the 14 products intended for children over 1 year were 1.26 μg/g and 31.85 μg/g, respectively. Some researchers have argued that all infant formulas should be “staged” to better meet the changing nutritional requirements of growing children [115]; such staging would require a reassessment of the growing child’s need for Mn from birth through the toddler years.

### Comparison of manganese concentrations in infant formulas and young child nutritional beverage products to Mn concentrations in breast milk and daily manganese intakes

It is presently assumed that breast milk from healthy, well-nourished mothers supplies adequate amounts of macro- and micro-nutrients, including Mn, at least for the first 6 months of life [54]. A review of longitudinal studies of Mn concentrations in breast milk reported average concentrations of 3–6 μg/L 2–4 weeks post-partum, with individual values between 2–8 μg/L [55]. A recent study of the Mn content in breast milk from various geographic areas (Argentina, Namibia, Poland, and the United States) reported mean values of 2–11 μg/L with individual values ranging from 1 to 30 μg/L [116]. All of the products that we tested contained substantially higher Mn concentrations (US minimum: 160 μg/L; France minimum: 200 μg/L) than the maximum concentration reported for breast milk. Assuming a mean value for breast milk around 3 μg/L in the US and France [116], minimal Mn concentrations in infant formula are about 53 (US) to 67 (France) times higher, while maximal Mn concentrations are 930 (US) and 400 (France) times higher. These ratios calculated from μg/L values are comparable with the ratios calculated from μg/100 kcal values (Fig 1). A substantially higher content of Mn in infant formula compared to breast milk has also been reported in all similar studies [56–58].

In a 2011 study of infant formulas in the Swedish market in which formulas were found to contain between 25–499 μg Mn/L, it was noted that daily Mn intakes of infants fed formula could be 114-fold higher than those of exclusively breast-fed infants [56]. The authors noted that “concentrations of several hundred μg/l, which we found in about half of the investigated formulas, may in fact not be safe for the infant” [56]. In the present study, in which the range of Mn content from formulas was 160–2800 μg Mn/L, daily intakes of Mn would be even higher than those discussed in the Swedish study [56].

### Infant formula and follow-on/follow-up formula standards and regulations

#### The joint World Health Organization/Food and Agriculture Organization Codex Alimentarius Commission standards for manganese in infant formula and follow-up formula

The joint WHO/FAO Codex Alimentarius Commission (CAC) publishes standards for infant formulas, follow-up formulas and formulas for special medical purposes intended for infants [88,89]. For infant formula nutrients such as Mn, standards are stated in mass of nutrient/100 kilocalorie (kcal) of prepared formula to automatically scale to infant energy requirements according to infant body weight, to varying types of formula (powder or liquid), and to varying masses of powder used to prepare a volume of formula. The CAC states that infant formula prepared for consumption shall contain between 60 kcal (250 kJ) and 70 kcal (295 kJ) of energy per 100 mL, a minimum of 1 μg of Mn/100 kcal, and not exceed the Guidance Upper Level (GUL) of 100 μg of Mn/100 kcal [2, 88]. The CAC does not provide guidance for the Mn content of follow-on formulas [89].

In this study, the mean energy content of the 42 powdered products was 683._7…_ kcal/L of prepared formula; therefore, the 1 μg of Mn/100 kcal minimum equals 6.84 μg of Mn/L of prepared product in this study as follows (see Eq 2; nonsignificant digits, such as 7…, are shown as a subscript followed by an ellipsis and are included in all steps of a calculation to prevent rounding error).

$$\begin{array}{c} Estimated\;Minimum\\ Volume\;Basis \end{array}=\frac{1\;\mu g\;of\;Mn}{100\;kcal}\times \frac{683._{7\ldots }kcal}{L\;of\;prepared\;product}=\frac{6.84\;\mu g\;of\;Mn}{L\;of\;prepared\;product}$$ (2)

Similarly, this 100 μg of Mn/100 kcal GUL equals 684 μg of Mn/L of prepared product in this study as follows (see Eq 3).

$$\begin{array}{c} Estimated\;Guidance\\ Upper\;Level\\ Volume\;Basis \end{array}=\frac{100\;\mu g\;of\;Mn}{100\;kcal}\times \frac{683._{7\ldots }kcal}{\begin{array}{c} L\;of\;prepared\\ product \end{array}}=\frac{684\;\mu g\;of\;Mn}{\begin{array}{c} L\;of\;prepared\\ product \end{array}}$$ (3)

None of the 17 powdered infant or follow-on formulas purchased in France for this study had a Mn concentration greater than the CAC 100 μg of Mn/100 kcal GUL. One of the 2 liquid products, an infant complementary food, had a concentration of 140 μg of Mn/100 kcal. Complementary foods are not covered by the CAC Standard for Follow-Up Formula [89]. This sample was not supplemented with Mn but contained chocolate, which can have a large effect on the concentration of Mn (Fig 1 and Table 3) [98,104–107].

Two of the 16 infant formula products purchased in the US for this study had a Mn concentration that was greater than the CAC 100 μg of Mn/100 kcal GUL for infant formulas. One of these products was a soy-based infant formula and the other was an amino acid-based medical infant formula. Five other products exceeded 100 μg of Mn/100 kcal but were labeled for children ages 12 months and older; the CAC standard does not stipulate a maximum Mn content for follow-up formulas [89]. The concentrations of Mn in the 7 US products with more than 100 μg of Mn/100 kcal ranged from 120 to 860 μg of Mn/100 kcal. Five of the 7 products that exceeded 100 μg of Mn/100 kcal contained supplemental Mn. Of the 2 products that exceeded 100 μg of Mn/100 kcal and did not contain supplemental Mn, 1 was soy-based and the other contained chocolate. These ingredients can have a large effect on the concentration of Mn (Fig 1 and Table 3) [93,98,104–107].

Three of the 7 products from the US market that exceeded the CAC 100 μg of Mn/100 kcal GUL for infant formula had “toddler” formula or powder on the manufacturer’s label, not “infant formula”. Similar to follow-up formulas, toddler formulas and toddler powders are marketed as milk substitutes for young children, but are not regulated as infant formulas, and their status with respect to the CAC standards is unclear [113,114,117,118]. The CAC states that “Follow-up formula is a food prepared from the milk of cows or other animals and/or other constituents of animal and/or plant origin, which have been proved to be suitable for infants from the 6th month on and for young children”, where young children are defined as “persons from the age of more than 12 months up to the age of three years (36 months)” [89]. By these CAC definitions, products labelled “toddler formula” would be classified as “follow-up” formulas, except they have not “been proved to be suitable for infants from the 6th month on and for young children” [89]. Terms such as “toddler formula”, “toddler powder”, or “toddler beverage” on labels may suggest to parents that these beverages can be used similar to infant or follow-on formulas [113,114,117,118]. Moreover, the WHO has observed, “It is clear that the marketing of toddler milks is a response to legislation that restricts marketing of formulas to infants” and emphasizes “the now common cross-promotion practice by which breast-milk substitutes for infants are promoted through labelling and advertisements of toddler formulas is a threat to breastfeeding and infant health” [118].

#### The European Union (EU) and the Republic of France regulations for manganese in infant formula and follow-on formula

The European Parliament regulates infant formulas and follow-on formulas within the European Union [9,90] while the the *République Française* (Republic of France) publishes regulations covering *préparations pour nourrissons et aux préparations de suite* (infant formulas and follow-on formulas) in France [3]. The current EU and French regulations for infant and follow-on formulas stipulate a minimum content of 1 μg of Mn/100 kcal and a maximum content of 100 μg of Mn/100 kcal [3,9].

In this study, all of the 17 infant or follow-on formulas purchased in France contained more than 1 μg of Mn/100 kcal of prepared product. None of the infant or follow-on formulas purchased in France had a measured Mn concentration that was greater than the 100 μg of Mn/100 kcal Maximum allowed by French law [3].

The concentrations of Mn in the 17 infant or follow-on formulas purchased in France ranged from 32 to 93 μg of Mn/100 kcal. The product with 93 μg of Mn/100 kcal has about 110 times more Mn than breast milk [92]. This product contained supplemental Mn. The supplementation of infant formula with Mn is allowed by the Republic of France [3]. In this study, 17 of the 19 products that we purchased in France were supplemented with Mn. The list of *sels autorisés* (allowed salts) is manganese carbonate, manganese chloride, manganese citrate, manganese sulfate, and manganese gluconate [3]. In 2002, the *Agence française de sécurité sanitaire des aliments* (AFSSA; French Food Safety Agency) stated “*l’enrichissement d’une préparation de suite en manganèse n’a aucune justification nutritionnelle* (the enrichment of follow-on formula with manganese does not have any nutritional justification)” [119].

#### The Food and Drug Administration and United States nutritional standards for manganese in infant formula

The Federal Food, Drug, and Cosmetic Act passed by the US Congress regulates infant formulas in the US [1]. According to the Federal Food, Drug, and Cosmetic Act, the Minimum Level for Mn in prepared infant formula is 5 μg/100 kcal, and no Maximum Level is specified [1].

In the 42 powdered products that we tested, which had a mean energy content of 683._7…_ kcal/L of prepared product, 5 μg of Mn/100 kcal would correspond to approximately 34 μg of Mn/L when prepared according to labelled instructions (see Eq 4).

$$\begin{array}{c} Estimated\\ Minimum\;Level\\ Volume\;Basis \end{array}=\frac{5\;\mu g\;of\;Mn}{100\;kcal}\times \frac{683._{7\ldots }kcal}{L\;of\;prepared\;product}=\frac{34\;\mu g\;of\;Mn}{L\;of\;prepared\;product}$$ (4)

All of the products (25 out of 25) purchased in the US for this study had a measured Mn concentration that was greater than the 5 μg of Mn/100 kcal FDA Minimum Level for infant formulas. The concentrations of Mn in these 25 products ranged from 26 to 860 μg of Mn/100 kcal. The product with 860 μg of Mn/100 kcal has about 1,000 times more Mn than breast milk [92]. This product was labeled “toddler powder”, so it is not regulated by US laws regarding infant formula [113,114].

Sixty-eight percent (17 out of 25) of the products we purchased on the US market were supplemented with Mn. In 1985, manganese chloride, manganese citrate, manganese gluconate, and manganese sulfate were “generally recognized as safe (GRAS) as a direct human food ingredient” and approved as sources of Mn for use in “infant formulas in accordance with section 412(g) of the Federal Food, Drug, and Cosmetic Act” [120]. By definition, GRAS is “A food substance that is not subject to premarket review and approval by FDA because it is generally recognized, by qualified experts, to be safe under the intended conditions of use” [121].

### Study limitations

By design, the maximum variation sampling method for this study focused on the margins of the markets, to identify products that might potentially have either too little or too much manganese to satisfy regulatory requirements. Consequently, no inferential statistics can be made about the markets as a whole from our results, and the only statistics that can be compared across the French and US markets are maxima and minima; measures of central tendency, sample variation, and tests of statistical significance concerning market differences cannot be performed with these data. In addition, our selection of products according to maximum variation sampling relied on labeled Mn content and ingredients such as soy, rice, cow milk, goat milk, and chocolate, since we predicted these factors to influence Mn content; however, actual Mn content may not be accurately labeled or may be determined by other factors. For this reason, we included multiple examples of each type of product that we predicted were likely to have very high or very low Mn content. Widespread Mn supplementation of products limited our efforts to determine whether specific ingredients such as soy protein, rice, cow milk, goat milk, or chocolate might be associated with high or low Mn content since it was difficult to find sufficient numbers of unsupplemented products of specific types. Furthermore, this study considers only whether products meet regulatory requirements and guidelines; it does not directly examine the question of whether feeding with these products would lead to either adequate or excess intakes of Mn, or the link between high Mn intake in children and brain disorder and adverse neurodevelopmental effects.

## Conclusions

In this study we used simultaneous particle induced X-ray emission (PIXE) and Rutherford backscattering (RBS) spectrometry to measure the concentration of Mn in μg of element per g of dried product for a selection of infant formulas and young child nutritional beverage products purchased in the US and France. The accuracy of PIXE/RBS for measuring Mn was assessed by the analysis of a National Institute of Standards & Technology (NIST) Standard Reference Material®, 1849a Infant/Adult Nutritional Formula; the relative error for the measurements of Mn was -0.22%. All 44 of the samples we analyzed had measurable concentrations of Mn.

In general, products with supplemental Mn had higher Mn concentrations than products without supplemental Mn (Fig 1 and Table 3). For products without supplemental Mn, products with chocolate had the most Mn, followed by soy, rice, and cow milk, all of which had more Mn than reported concentrations in breast milk (Fig 1 and Table 3).

The ranges of concentrations of Mn in the infant formulas and young child nutritional beverage products greatly exceeded the ranges of Mn concentrations reported in breast milk. The ranges of Mn concentrations in the 17 infant and follow-on formula products purchased in France were in compliance with French, European, and international standards. The ranges of Mn concentrations in the 25 infant formula and young child nutritional beverages purchased in the US satisfied the US minimum standards for infant formula. While 2 of the 16 US infant formula products and 5 of the 8 products labeled for children over 12 months that we tested exceeded the CAC international Guidance Upper Level (GUL) of 100 Mn/100 kcal for infant formulas, US laws do not currently stipulate a maximum Mn content for infant formulas or regulate Mn content in formulas or powders for children over 12 months old and the CAC does not stipulate a GUL for Mn for formula products for children over 12 months old 89].

Given the recent research demonstrating adverse effects of excess Mn exposure for neurodevelopment [4–7,10–47], stricter upper limits for Mn content in infant formulas should be considered by regulators. The 38-year-old requirement for minimum Mn content in infant formulas in the US [1] may need to be updated to make it closer to the Mn content in breast milk, and a maximum Mn content for infant formulas and young child nutritional beverage products may be appropriate for the US market.

In the meantime, it must be noted that supplementation of infant formulas and young child nutritional beverage products with Mn is unnecessary to meet the current health-based regulatory minimum of 1 μg Mn/100 kcal stipulated by the CAC, EU and Republic of France, or the US minimum of 5 μg Mn/100 kcal [1–3,9,90]. There is no proven or likely benefit from Mn supplementation in these products and some researchers contend there is potential for adverse neurodevelopmental health effects in infants and young children. Pending research on dietary Mn exposures that examine health effects at high and low Mn dietary intakes in neonates, infants, and young children, formula manufacturers may consider taking measures to reduce Mn content in their products to approach the Mn concentrations found in breast milk rather than supplement their products with additional Mn when the products already have much higher Mn concentrations than breast milk.

## Supporting information

S1 Table Product label information. (DOCX)

S2 Table Reconstitution according to labels only. (DOCX)

S3 Table Labelled energy content. (DOCX)

S4 Table Labeled Mn content. (DOCX)

S5 Table Laboratory reconstitution measurements of powdered samples. (DOCX)

S6 Table Water and powder in 1 L of prepared formula. (DOCX)

S7 Table Mass of solids in 1 L of liquid samples (by evaporation starting with measured volumes). (DOCX)

S8 Table Calculations to derive g powder (or solids) / 100 kCal based on laboratory measurements of g powder (or solids) / L prepared formula and labeled energy content. (DOCX)

S9 Table PIXE data. (DOCX)

S10 Table Mean NIST measurements. (DOCX)

## Abbreviations

μg: microgram
μL: microliter
μm: micrometer
^241^Am: the 241 isotope of americium
C: Celsius
Ca: calcium
CAC: Codex Alimentarius Commission
CENBG: Centre d’Etudes Nucléaires de Bordeaux Gradignan
CFR: Code of Federal Regulations
Cl: chlorine
cm: centimeter
CNRS: Centre National de la Recherche Scientifique
Cu: copper
e^−^: electron
EC: European Commission
EU: European Union
eV: electron volt
FAO: Food and Agriculture Organization
FDA: Food and Drug Administration
Fe: iron
FWHM: full width at half maximum
g: gram
GRAS: generally recognized as safe
GUL: Guidance Upper Level
H^+^: proton
IN2P3: Institut National de Physique Nucléaire et de Physique des Particules
K: potassium
kcal: kilocalorie
keV: kiloelectron volt
kg: kilogram
kj: kilojoule
L: liter
Max: maximum
MeV: megaelectron volt
mg: milligram
Min: minimum
mL: milliliter
mm^2^: square millimeter
Mn: manganese
MR: magnetic resonance
N: nitrogen
n: number of samples
Na: sodium
NIST: National Institute of Standards & Technology
O: oxygen
P: phosphorus
pA: picoampere
PIXE: particle induced X-ray emission
Rb: rubidium
RBS: Rutherford backscattering spectrometry
S: sulfur
s: sample standard deviation
Si: silicon
Si(Li): lithium-drifted silicon x-ray detector
Sr: strontium
SRM: Standard Reference Material
US: United States
WHO: World Health Organization
$\bar{x}$: sample mean
Zn: zinc
