# Supplementary material for: Manganese levels in infant formula and young child nutritional beverages in the United States and France: Comparison to breast milk and regulations
Source: PLoS One. 2019 Nov 5;14(11):e0223636. doi: 10.1371/journal.pone.0223636 (PMC6830775; doi:10.1371/journal.pone.0223636)
Supplement: S1 Table — (DOCX) [file pone.0223636.s001.docx]

**S1. Product label information**

| **Sample Number** | **Age Description** | **Code on container** | **Organic** | **Container material(s)** | **Protein Source** | **Chocolate** | **Supplemental Mn** | **Product Name** |
| --- | --- | --- | --- | --- | --- | --- | --- | --- |
| **FR01** | 0-6 months | 1 |  | aluminum-wrapped cardboard | cow milk |  | yes | Blédina Blédilait Lait 1er Age en Poudre de Naissance à 6 Mois |
| **FR02** | 12+ months | 3 |  | aluminum-wrapped cardboard | cow milk |  | yes | Laboratoire Gallia Lait de Croissance dès 12 Mois Calisma |
| **FR03** | 6-12 months | 2 |  | aluminum | cow milk |  | yes | Laboratoire Gallia Calisma Lait 2Ème Âge en Poudre de 6 à 12 Mois |
| **FR04** | 6+ months | 2 | yes | aluminum | goat milk |  | yes | Holle bio le lait de suite 2 de lait de chèvre |
| **FR05** | 1+ years | 3 |  | metal | goat milk |  | yes | CapriCare Lait de Chèvre 3ème âge |
| **FR06** | 0-6 months | 1 |  | metal | cow milk |  |  | GUIGOZ Lait Evolia Relais Allaitement 1e Âge |
| **FR07** | 0-6 months | 1 |  | foil | cow milk |  | yes | Nestlé Nidal 1 - Lait Infantile 1er Âge en Poudre - de la Naissance à 6 Mois |
| **FR08** | 6+ months | 2 |  | sterilepak | cow milk | yes |  | Nestlé Bébé P'tit Dej Saveur Chocolat au lait - Brique Lait & Céréales dès 6 Mois |
| **FR09** | 6+ months | 2 |  | sterilepak | cow milk |  |  | Nestlé Bébé P'tit Souper Lait Carottes Riz - Soupe du soir dès 6 Mois |
| **FR10** | 6+ months | 2 |  | metal | goat milk |  | yes | Lait en Poudre Kabrita Goats Infant Milk Stage |
| **FR11** | 6-12 months | 2 |  | metal | cow milk |  | yes | Laboratoires Picot AR anti-regurgitations 2 |
| **FR12** | 10+ months | 3 | yes | aluminum | cow milk |  | yes | Suite de Holle Bio Lait 3, 1er Pack |
| **FR13** | 1-3 years | 3 |  | metal | cow milk |  | yes | Sma Lait Enfant 1-3 Ans Poudre |
| **FR14** | 6-12 months | 2 |  | aluminum-wrapped cardboard | cow milk |  | yes | MODILAC Lait Expert Doucea 2° âge |
| **FR15** | 6 months + | 2 | yes | aluminum-wrapped cardboard | cow milk |  | yes | Hipp Biologique Du Lait De Bonne Nuit Partir De 6 Mois |
| **FR16** | 6-12 months | 2 |  | aluminum-wrapped cardboard | cow milk |  | yes | Cow & Gate Follow-on Milk |
| **FR17** | 1+ years | 3 | yes | aluminum | cow milk |  | yes | Hipp Combiotic Organique Lait De Croissance 3 À Partir Du 12E Mois À Compter |
| **FR18** | 12+ months | 4 | yes | aluminum | cow milk |  | yes | Holle Bio Lait Enfant 4 |
| **FR19** | 0-36 months | 1 |  | aluminum-wrapped cardboard | cow milk |  | yes | Lait en poudre Novalac ad en cas de diarrhée |
| **US01** | infant |  |  | aluminum-wrapped cardboard | cow milk |  |  | Similac Sensitive for Spit-Up Powder |
| **US02** | 6 months + | 2 |  | aluminum | cow milk |  |  | Malutka Infant Baby Formula With Prebiotics and Nucleotides and Rice Flour Milk and Cereal Based Dry Babies Formula non-GMO from 6 Month Babies |
| **US03** | 0-12 months | |  | aluminum | cow milk |  | yes | Enfamil A.R. Infant Formula for Spit Up, Powder |
| **US04** | toddler |  | yes | aluminum-wrapped cardboard | cow milk |  |  | Baby's Only Organic Dairy Formula |
| **US05** | toddler |  | yes | aluminum-wrapped cardboard | soy protein |  |  | Babys Only Soy Organic Toddler Formula |
| **US06** | 0-12 months | |  | plastic | cow milk |  | yes | Gerber Good Start Soothe Non-GMO Powder Infant Formula, Stage 1 |
| **US07** | infant |  |  | plastic | cow milk |  | yes | Target Infant Formula, Added Rice Starch |
| **US08** | 1+ years |  |  | aluminum-wrapped cardboard | amino acids |  | yes | Neocate Junior, Chocolate |
| **US09** | 1-3 years |  |  | metal | goat milk |  | yes | Nannycare Nanny Goat Milk Growing Up Nutrition |
| **US10** | 1-13 years |  | yes | aluminum-wrapped cardboard | cow milk | yes | yes | PediaSmart Organic DAIRY Chocolate Complete Nutrition Beverage Powder |
| **US11** | 13 months-8 years | | yes | plastic | goat milk | yes |  | Perfectly Healthy Toddler Goat Milk Formula Chocolate |
| **US12** | 1-4 years |  | yes | aluminum-wrapped cardboard | rice protein |  | yes | VegLife Toddler Supreme Rice Protein Powder, Vanilla |
| **US13** | 1-2 years |  |  | metal | goat milk |  | yes | Kabrita Non-GMO Goat Milk Toddler Formula |
| **US14** | infant |  |  | aluminum-wrapped cardboard | amino acids |  | yes | EleCare For Infants Unflavored Powder with DHA/ARA |
| **US15** | infant |  |  | aluminum-wrapped cardboard | soy protein |  |  | Similac Soy Isomil Baby Formula - Powder |
| **US16** | infant |  |  | aluminum-wrapped cardboard | soy protein |  |  | Enfamil ProSobee Soy Infant Formula, Powder |
| **US17** | 0-12 months | |  | aluminum-wrapped cardboard | soy protein |  |  | Gerber Good Start Formula Soy Powder Stage 1 For Birth to 12 Months |
| **US18** | 0-12 months | |  | metal | amino acids |  | yes | Alfamino Infant Amino Acid Formula Powder |
| **US19** | 0-12 months | | yes | metal | soy protein |  | yes | Earth's Best Organic Soy Infant Formula with Iron |
| **US20** | infant |  |  | aluminum-wrapped cardboard | cow milk |  | yes | Similac Advance Baby Formula - Powder |
| **US21** | infant |  |  | aluminum-wrapped cardboard | cow milk |  | yes | Enfamil Infant Formula, Powder |
| **US22** | infant |  |  | aluminum-wrapped cardboard | cow milk |  | yes | Gerber Good Start Gentle for Supplementing Baby Formula - Powder |
| **US23** | infant |  |  | plastic | cow milk |  | yes | GoodSense Advantage Non-GMO Milk-Based Powder Infant Formula with Iron |
| **US24** | infant |  |  | plastic | cow milk |  | yes | GoodSense Gentle Milk-Based Powder Infant Formula with Iron |
| **US25** | infant |  |  | aluminum-wrapped cardboard | cow milk |  | yes | Enfamil Gentlease Gentle Infant Formula, Powder |

All products were purchased from September 6 to September 19, 2017.

All French market samples (F01-F19) were purchased from Amazon.fr.

All US market samples (U01-U25) were purchased from Amazon.com.
