# Supplementary material for: Manganese levels in infant formula and young child nutritional beverages in the United States and France: Comparison to breast milk and regulations
Source: PLoS One. 2019 Nov 5;14(11):e0223636. doi: 10.1371/journal.pone.0223636 (PMC6830775; doi:10.1371/journal.pone.0223636)
Supplement: S2 Table — (DOCX) [file pone.0223636.s002.docx]

**S2. Reconstitution according to labels only**

*By US labeling law, 1 oz = 30 mL (21 CFR 101.9, 2017)*

*Unless a correction for powder addition is specifically noted on label, stated batch size = volume of water added / batch. See "Lab reconstitution" worksheet for reconstitution data measured by us.*

| **Color key** |  | *=as stated on label* |
| --- | --- | --- |
|  |  | *=calculated by us based on labeled information* |

| **Sample Number** | **Labeled Scoop size (g)** | **# of scoops / batch (scoops)** | **Water to add / batch (oz)** | **Water to add / batch (mL)** | **Stated batch size (oz)** | **Stated batch size (mL)** | **Stated Reconstitution %** | **g powder / batch (g)** | **g powder / mL prepared product (by instructions) (g)** | **g powder / 100 mL prepared product (by instructions) (g)** | **g powder / L prepared product (by instructions) (g)** | **L prepared product / container (by instructions) (L)** |  |  |  |  |
| --- | --- | --- | --- | --- | --- | --- | --- | --- | --- | --- | --- | --- | --- | --- | --- | --- |
| **FR01** |  | 1 |  | 30 |  |  | 13.6% |  | 0.136 | 13.6 | 136 | 6.617647059 |  |  |  |  |
| **FR02** |  | 1 |  | 30 |  |  | 14.4% |  | 0.144 | 14.4 | 144 | 6.25 |  |  |  |  |
| **FR03** |  | 1 |  | 30 |  |  | 14.2% |  | 0.142 | 14.2 | 142 | 8.450704225 |  |  |  |  |
| **FR04** | 4.5 | 5 |  | 150 |  | 170 | 13.2% | 22.5 | 0.132352941 | 13.23529412 | 132.3529412 | 3.022222222 |  |  |  |  |
| **FR05** | 4.3 | 3 |  | 90 |  | 100 | 12.9% | 12.9 | 0.129 | 12.9 | 129 | 3.100775194 |  |  |  |  |
| **FR06** | 4.3 | 3 |  | 90 |  |  | 13.0% |  | 0.13 | 13 | 130 | 6.153846154 |  |  |  |  |
| **FR07** | 4.3 | 3 |  | 90 |  |  | 12.9% | 12.9 | 0.129 | 12.9 | 129 | 5.426356589 |  |  |  |  |
| **FR08** |  |  |  |  |  | 500 |  |  |  |  |  | 2 |  |  |  |  |
| **FR09** |  |  |  |  |  | 500 |  |  |  |  |  | 2 |  |  |  |  |
| **FR10** | 4.666667 | 3 |  | 90 |  | 100 | 14.0% | 14 | 0.14 | 14 | 140 | 5.714285714 |  |  |  |  |
| **FR11** | 4.5 | 7 |  | 210 |  |  | 13.5% | 31.5 | 0.135 | 13.5 | 135 | 6.666666667 |  |  |  |  |
| **FR12** | 4.7 | 5 |  | 150 |  | 170 | 13.8% | 23.5 | 0.138235294 | 13.82352941 | 138.2352941 | 4.340425532 |  |  |  |  |
| **FR13** | 4.2 | 6 |  | 180 |  | 200 | 12.6% | 25.2 | 0.126 | 12.6 | 126 | 3.174603175 |  |  |  |  |
| **FR14** | 4.8 | 1 |  | 30 |  |  | 14.3% | 4.8 | 0.143 | 14.3 | 143 | 5.594405594 |  |  |  |  |
| **FR15** | 5 | 5 |  | 90 |  | 100 | 14.0% | 25 | 0.14 | 14 | 140 | 2.5 |  |  |  |  |
| **FR16** | 4.9 | 1 | 1 | 30 |  | 30 | 16.3% | 4.9 | 0.163333333 | 16.33333333 | 163.3333333 | 5.510204082 |  |  |  |  |
| **FR17** | 4.6 | 5 | 5 | 150 |  | 150 | 15.3% | 23 | 0.153333333 | 15.33333333 | 153.3333333 | 3.913043478 |  |  |  |  |
| **FR18** | 4.7 | 5 |  | 150 |  | 170 | 13.8% | 23.5 | 0.138235294 | 13.82352941 | 138.2352941 | 4.340425532 |  |  |  |  |
| **FR19** | 4.3 | 1 |  | 30 |  | 30 | 13.0% | 4.3 | 0.13 | 13 | 130 | 4.615384615 |  |  |  |  |
| **US01** | 8.3 | 1 | 2 | 60 | 2 | 60 | 13.8% | 8.3 | 0.138333333 | 13.83333333 | 138.3333333 | 2.457831325 |  |  |  |  |
| **US02** | 4.5 | 6 |  | 180 |  | 200 | 13.5% | 27 | 0.135 | 13.5 | 135 | 2.592592593 |  |  |  |  |
| **US03** | 9 | 1 | 2 | 60 | 2 | 60 | 15.0% | 9 | 0.15 | 15 | 150 | 2.44 |  |  |  |  |
| **US04** | 8.5 | 1 | 2 | 60 | 2 | 60 | 14.2% | 8.5 | 0.141666667 | 14.16666667 | 141.6666667 | 2.470588235 |  |  |  |  |
| **US05** | 8.75 | 1 | 2 | 60 | 2 | 60 | 14.6% | 8.75 | 0.145833333 | 14.58333333 | 145.8333333 | 2.4 |  |  |  |  |
| **US06** | 8.7 | 1 | 2 | 60 | 2.2 | 66 | 13.2% | 8.7 | 0.131818182 | 13.18181818 | 131.8181818 | 4.779310345 |  |  |  |  |
| **US07** | 8.9 | 1 | 2 | 60 | 2 | 60 | 14.8% | 8.9 | 0.148333333 | 14.83333333 | 148.3333333 | 4.112359551 |  |  |  |  |
| **US08** | 8.2 | 4 | 4 | 120 | 4 | 120 | 27.3% | 32.8 | 0.273333333 | 27.33333333 | 273.3333333 | 1.463414634 |  |  |  |  |
| **US09** | 4.5 | 1 |  | 30 | 1.1 | 33 | 13.6% | 4.5 | 0.136363636 | 13.63636364 | 136.3636364 | 2.933333333 |  |  |  |  |
| **US10** | 17 | 3 | 7 | 210 | 8 | 210 | 24.3% | 51 | 0.242857143 | 24.28571429 | 242.8571429 | 1.441176471 |  |  |  |  |
| **US11** | 20 | 2 | 8 | 240 | 8 | 240 | 16.7% | 40 | 0.166666667 | 16.66666667 | 166.6666667 | 2.7 |  |  |  |  |
| **US12** | 13.5 | 2 | 8 | 240 | 8 | 240 | 11.3% | 27 | 0.1125 | 11.25 | 112.5 | 3.528888889 |  |  |  |  |
| **US13** | 4.8 | 2 | 2 | 60 | 2 | 60 | 16.0% | 9.6 | 0.16 | 16 | 160 | 2.5 |  |  |  |  |
| **US14** | 9.4 | 1 | 2 | 60 | 2 | 60 | 15.7% | 9.4 | 0.156666667 | 15.66666667 | 156.6666667 | 2.553191489 |  |  |  |  |
| **US15** | 8.8 | 1 | 2 | 60 | 2 | 60 | 14.7% | 8.8 | 0.146666667 | 14.66666667 | 146.6666667 | 2.386363636 |  |  |  |  |
| **US16** | 8.8 | 1 | 2 | 60 | 2.2 | 66 | 13.3% | 8.8 | 0.133333333 | 13.33333333 | 133.3333333 | 2.745 |  |  |  |  |
| **US17** | 8.9 | 1 | 2 | 60 | 2 | 60 | 14.8% | 8.9 | 0.148333333 | 14.83333333 | 148.3333333 | 2.46741573 |  |  |  |  |
| **US18** | 4.7 | 2 | 2 | 60 | 2 | 60 | 15.7% | 9.4 | 0.156666667 | 15.66666667 | 156.6666667 | 2.553191489 |  |  |  |  |
| **US19** | 8.8 | 1 | 2 | 60 | 2.2 | 66 | 13.3% | 8.8 | 0.133333333 | 13.33333333 | 133.3333333 | 4.875 |  |  |  |  |
| **US20** | 8.7 | 1 | 2 | 60 | 2 | 60 | 14.5% | 8.7 | 0.145 | 14.5 | 145 | 2.413793103 |  |  |  |  |
| **US21** | 8.8 | 1 | 2 | 60 | 2 | 60 | 14.7% | 8.8 | 0.146666667 | 14.66666667 | 146.6666667 | 2.386363636 |  |  |  |  |
| **US22** | 8.7 | 1 | 2 | 60 | 2 | 60 | 14.5% | 8.7 | 0.145 | 14.5 | 145 | 2.413793103 |  |  |  |  |
| **US23** | 8.7 | 1 | 2 | 60 | 2 | 60 | 14.5% | 8.7 | 0.145 | 14.5 | 145 | 4.482758621 |  |  |  |  |
| **US24** | 8.8 | 1 | 2 | 60 | 2 | 60 | 14.7% | 8.8 | 0.146666667 | 14.66666667 | 146.6666667 | 4.159090909 |  |  |  |  |
| **US25** | 8.7 | 1 | 2 | 60 | 2 | 60 | 14.5% | 8.7 | 0.145 | 14.5 | 145 | 2.413793103 |  |  |  |  |
