# Supplementary material for: Manganese levels in infant formula and young child nutritional beverages in the United States and France: Comparison to breast milk and regulations
Source: PLoS One. 2019 Nov 5;14(11):e0223636. doi: 10.1371/journal.pone.0223636 (PMC6830775; doi:10.1371/journal.pone.0223636)
Supplement: S3 Table — (DOCX) [file pone.0223636.s003.docx]

**S3. Labelled energy content**

*By US labeling law, 1 "Calorie" = 1 kCal*

*By US labeling law, 1 oz = 30 mL (21 CFR 101.9, 2017)*

| **Color key** |  | *=as stated on label* |
| --- | --- | --- |
|  |  | *=calculated by us based on labeled information* |

| **Sample Number** | **"Calories" / Serving (kCal)** | **Serving size (oz)** | **Serving size (mL)** | **Servings / batch (servings)** | **Serving size (g powder)** | **kCal / 100 g powder (kCal)** | **kCal / g powder (kCal)** | **g powder / kCal (g)** | **g powder / 100 kCal (g)** | **kCal / 100 mL prepared formula** |  |  |  |  |
| --- | --- | --- | --- | --- | --- | --- | --- | --- | --- | --- | --- | --- | --- | --- |
| **FR01** |  |  |  |  |  | 484 | 4.84 | 0.206612 | 20.66115702 | 65.824 |  |  |  |  |
| **FR02** |  |  |  |  |  | 451 | 4.51 | 0.221729 | 22.172949 | 64.944 |  |  |  |  |
| **FR03** |  |  |  |  |  | 467 | 4.67 | 0.214133 | 21.41327623 | 66.314 |  |  |  |  |
| **FR04** |  |  |  |  |  | 496 | 4.96 | 0.201613 | 20.16129032 | 65.64706 |  |  |  |  |
| **FR05** |  |  |  |  |  | 506 | 5.06 | 0.197628 | 19.76284585 | 65.274 |  |  |  |  |
| **FR06** |  |  |  |  |  | 515 | 5.15 | 0.194175 | 19.41747573 | 66.95 |  |  |  |  |
| **FR07** |  |  |  |  |  | 517 | 5.17 | 0.193424 | 19.34235977 | 66.693 |  |  |  |  |
| **FR08** |  |  |  |  |  |  |  |  |  | 86 |  |  |  |  |
| **FR09** |  |  |  |  |  |  |  |  |  | 84 |  |  |  |  |
| **FR10** |  |  |  |  |  | 478.5714 | 4.785714 | 0.208955 | 20.89552239 | 67 |  |  |  |  |
| **FR11** |  |  |  |  |  | 483 | 4.83 | 0.207039 | 20.70393375 | 65.205 |  |  |  |  |
| **FR12** |  |  |  |  |  | 484 | 4.84 | 0.206612 | 20.66115702 | 66.90588 |  |  |  |  |
| **FR13** |  |  |  |  |  | 507.9365 | 5.079365 | 0.196875 | 19.6875 | 64 |  |  |  |  |
| **FR14** |  |  |  |  |  | 480 | 4.8 | 0.208333 | 20.83333333 | 68.64 |  |  |  |  |
| **FR15** |  |  |  |  |  | 500 | 5 | 0.2 | 20 | 70 |  |  |  |  |
| **FR16** |  |  |  |  |  | 451.449 | 4.51449 | 0.221509 | 22.1508972 | 68 |  |  |  |  |
| **FR17** |  |  |  |  |  | 466.7474 | 4.667474 | 0.214249 | 21.42486284 | 66 |  |  |  |  |
| **FR18** |  |  |  |  |  | 481 | 4.81 | 0.2079 | 20.79002079 | 66.49118 |  |  |  |  |
| **FR19** |  |  |  |  |  | 477.4 | 4.774 | 0.209468 | 20.94679514 | 62.062 |  |  |  |  |
| **US01** | 100 | 5.3 | 159 | 2.65 | 21.995 | 500.1137 | 5.001137 | 0.199955 | 19.99545455 | 69.18239 |  |  |  |  |
| **US02** |  |  |  |  |  | 496.5 | 4.965 | 0.20141 | 20.14098691 | 67.0275 |  |  |  |  |
| **US03** | 100 | 5 | 150 | 2.5 | 22.5 | 488.8889 | 4.888889 | 0.204545 | 20.45454545 | 73.33333 |  |  |  |  |
| **US04** | 80 | 4 | 120 | 2 | 17 | 477.3744 | 4.773744 | 0.209479 | 20.9479168 | 67.62805 |  |  |  |  |
| **US05** | 80 | 4 | 120 | 2 | 17.5 | 463.7352 | 4.637352 | 0.21564 | 21.564032 | 67.62805 |  |  |  |  |
| **US06** | 100 | 5 | 150 | 2.2727273 | 19.77273 | 505.7471 | 5.057471 | 0.197727 | 19.77272727 | 66.66667 |  |  |  |  |
| **US07** | 100 | 5 | 150 | 2.5 | 22.25 | 487.354 | 4.87354 | 0.20519 | 20.51896375 | 72.29085 |  |  |  |  |
| **US08** | 100 |  | 100 |  |  | 451 | 4.51 | 0.221729 | 22.172949 | 123.2733 |  |  |  |  |
| **US09** |  | 6 | 180 | 5.4545455 | 27 | 484.3487 | 4.843487 | 0.206463 | 20.64628249 | 67 |  |  |  |  |
| **US10** | 240 | 8 | 240 | 1 | 52 | 510.2884 | 5.102884 | 0.195968 | 19.59676313 | 123.9272 |  |  |  |  |
| **US11** | 170 | 8 | 240 | 1 | 40 | 425 | 4.25 | 0.235294 | 23.52941176 | 70.83333 |  |  |  |  |
| **US12** | 100 | 8 | 240 | 1 | 27 | 370.3704 | 3.703704 | 0.27 | 27 | 41.66667 |  |  |  |  |
| **US13** | 90 | 4 | 120 | 2 | 19.2 | 508.295 | 5.08295 | 0.196736 | 19.67361318 | 81.32721 |  |  |  |  |
| **US14** | 100 | 5 | 150 | 2.5 | 23.5 | 475 | 4.75 | 0.210526 | 21.05263158 | 74.41667 |  |  |  |  |
| **US15** | 100 | 5 | 150 | 2.5 | 22 | 500 | 5 | 0.2 | 20 | 73.33333 |  |  |  |  |
| **US16** | 100 | 5 | 150 | 2.2727273 | 20 | 500 | 5 | 0.2 | 20 | 67.62805 |  |  |  |  |
| **US17** | 100 | 5 | 150 | 2.5 | 22.25 | 494.382 | 4.94382 | 0.202273 | 20.22727273 | 73.33333 |  |  |  |  |
| **US18** | 100 | 5 | 150 | 2.5 | 23.5 | 468.0851 | 4.680851 | 0.213636 | 21.36363636 | 73.33333 |  |  |  |  |
| **US19** | 100 | 5 | 150 | 2.2727273 | 20 | 492.8922 | 4.928922 | 0.202884 | 20.2884136 | 65.71895 |  |  |  |  |
| **US20** | 100 | 5 | 150 | 2.5 | 21.75 | 505.7471 | 5.057471 | 0.197727 | 19.77272727 | 73.33333 |  |  |  |  |
| **US21** | 100 | 5 | 150 | 2.5 | 22 | 500 | 5 | 0.2 | 20 | 73.33333 |  |  |  |  |
| **US22** | 100 | 5 | 150 | 2.5 | 21.75 | 505.7471 | 5.057471 | 0.197727 | 19.77272727 | 73.33333 |  |  |  |  |
| **US23** | 100 | 5 | 150 | 2.5 | 21.75 | 498.5576 | 4.985576 | 0.200579 | 20.05786344 | 72.29085 |  |  |  |  |
| **US24** | 100 | 5 | 150 | 2.5 | 22 | 492.8922 | 4.928922 | 0.202884 | 20.2884136 | 72.29085 |  |  |  |  |
| **US25** | 100 | 5 | 150 | 2.5 | 21.75 | 505.7471 | 5.057471 | 0.197727 | 19.77272727 | 73.33333 |  |  |  |  |
