# Supplementary material for: Manganese levels in infant formula and young child nutritional beverages in the United States and France: Comparison to breast milk and regulations
Source: PLoS One. 2019 Nov 5;14(11):e0223636. doi: 10.1371/journal.pone.0223636 (PMC6830775; doi:10.1371/journal.pone.0223636)
Supplement: S4 Table — (DOCX) [file pone.0223636.s004.docx]

**S4. Labeled Mn content**

*In the US, FDA labelling laws state total DV = 1.2 mg Mn / day for 1-3 year olds (*[*https://www.fda.gov/downloads/food/guidanceregulation/guidancedocumentsregulatoryinformation/labelingnutrition/ucm513817.pdf*](https://www.fda.gov/downloads/food/guidanceregulation/guidancedocumentsregulatoryinformation/labelingnutrition/ucm513817.pdf)*)*

| **Color key** |  | *=as stated on label* |
| --- | --- | --- |
|  |  | *=calculated by us based on labeled information* |

| **Sample Number** | **µg Mn / serving** | **%DV Mn / serving** | **µg Mn / 100 gm powder** | **µg Mn / 100 ml formula** | **µg Mn / 1 gm powder** | **µg Mn / L formula** | **µg Mn / 100 kCal** |
| --- | --- | --- | --- | --- | --- | --- | --- |
| **FR01** |  |  | 56 | 7.6 | 0.56 | 76 | 11.57025 |
| **FR02** |  |  | 54 | 7.8 | 0.54 | 78 | 11.97339 |
| **FR03** |  |  | 48 | 6.8 | 0.48 | 68 | 10.27837 |
| **FR04** |  |  | 92 | 12 | 0.92 | 120 | 18.54839 |
| **FR05** |  |  |  |  |  |  |  |
| **FR06** |  |  | 115 | 15 | 1.15 | 150 | 22.3301 |
| **FR07** |  |  | 125 | 16.2 | 1.25 | 162 | 24.17795 |
| **FR08** |  |  |  |  |  |  |  |
| **FR09** |  |  |  |  |  |  |  |
| **FR10** |  |  | 65 | 9.1 | 0.65 | 91 | 13.58209 |
| **FR11** |  |  | 56 | 7.6 | 0.56 | 76 | 11.6 |
| **FR12** |  |  | 110 | 15 | 1.1 | 150 | 22.72727 |
| **FR13** |  |  | 79.36508 | 10 | 0.793651 | 100 | 15.625 |
| **FR14** |  |  | 100 | 14.3 | 1 | 143 | 20.83333 |
| **FR15** |  |  | 164.2857 | 23 | 1.642857 | 230 | 32.85714 |
| **FR16** |  |  | 42.85714 | 7 | 0.428571 | 70 | 9.493242 |
| **FR17** |  |  | 42.3913 | 6.5 | 0.423913 | 65 | 9.082279 |
| **FR18** |  |  | 120 | 17 | 1.2 | 170 | 24.94802 |
| **FR19** |  |  | 200 | 26 | 2 | 260 | 41.9 |
| **US01** | 5 |  | 23.06026 | 3.190002 | 0.230603 | 31.90002 | 5 |
| **US02** | 45.9 |  | 170 | 22.95 | 1.7 | 229.5 | 34.23968 |
| **US03** | 15 |  | 67.62805 | 10.14421 | 0.67628 | 101.4421 | 15 |
| **US04** |  |  |  |  |  |  |  |
| **US05** |  |  |  |  |  |  |  |
| **US06** | 15 |  | 76.95605 | 10.14421 | 0.769561 | 101.4421 | 15 |
| **US07** | 15 |  | 67.41573 | 10 | 0.674157 | 100 | 15 |
| **US08** | 203.36 |  | 620 | 156.2823 | 6.2 | 1562.823 | 137.4723 |
| **US09** |  |  |  |  |  |  |  |
| **US10** | 240 | 20% | 686.2745 | 166.6667 | 6.862745 | 1666.667 | 166.6667 |
| **US11** |  |  |  |  |  |  |  |
| **US12** | 55 |  | 187.8557 | 21.13376 | 1.878557 | 211.3376 | 55 |
| **US13** |  |  |  |  |  |  |  |
| **US14** | 84 |  | 400 | 57.79124 | 4 | 577.9124 | 84.21053 |
| **US15** | 25 |  | 115.2751 | 16.90701 | 1.152751 | 169.0701 | 25 |
| **US16** | 25 |  | 126.8026 | 16.90701 | 1.268026 | 169.0701 | 25 |
| **US17** | 25 |  | 113.9799 | 16.90701 | 1.139799 | 169.0701 | 25 |
| **US18** | 15 |  | 64.75026 | 10.14421 | 0.647503 | 101.4421 | 15 |
| **US19** | 25 |  | 125 | 16.66667 | 1.25 | 166.6667 | 25 |
| **US20** | 5 |  | 23.32002 | 3.381402 | 0.2332 | 33.81402 | 5 |
| **US21** | 15 |  | 69.16505 | 10.14421 | 0.69165 | 101.4421 | 15 |
| **US22** | 15 |  | 69.96005 | 10.14421 | 0.6996 | 101.4421 | 15 |
| **US23** | 5 |  | 22.98851 | 3.333333 | 0.229885 | 33.33333 | 5 |
| **US24** | 15 |  | 68.18182 | 10 | 0.681818 | 100 | 15 |
| **US25** | 15 |  | 69.96005 | 10.14421 | 0.6996 | 101.4421 | 15 |
