# Supplementary material for: Manganese levels in infant formula and young child nutritional beverages in the United States and France: Comparison to breast milk and regulations
Source: PLoS One. 2019 Nov 5;14(11):e0223636. doi: 10.1371/journal.pone.0223636 (PMC6830775; doi:10.1371/journal.pone.0223636)
Supplement: S5 Table — (DOCX) [file pone.0223636.s005.docx]

**S5. Laboratory reconstitution measurements of powdered samples**

| **Sample Number** | **# of scoops measured** | **Mass of powder in 1 scoop--Rep 1 (g)** | **Mass of powder in 1 scoop--Rep 2 (g)** | **Mass of powder in 1 scoop--Rep 3 (g)** | **Average g powder / scoop (g)** | **Aliquot of water specified for 1 scoop of powder (mL)** | **Volume of water + powder--Rep 1 (mL)** | **Volume of water + powder--Rep 2 (mL)** | **Volume of water + powder--Rep 3 (mL)** | **Average volume of water + powder (mL)** | **Average g of powder / mL of prepared formula (g)** | **Average g of powder / 100 mL of prepared formula (g)** | **Added volume / aliquot water due to addition of 1 scoop powder (mL)** | **% added volume = % of prepared formula by volume that is powder** |
| --- | --- | --- | --- | --- | --- | --- | --- | --- | --- | --- | --- | --- | --- | --- |
| **FR01** | 1 | 4.6891 | 4.5286 | 4.4709 | 4.562867 | 30 | 33.2 | 33.2 | 33.1 | 33.16667 | 0.137573869 | 13.75738693 | 3.166666667 | 0.105555556 |
| **FR02** | 1 | 4.8161 | 4.6623 | 4.5184 | 4.6656 | 30 | 33.4 | 33.4 | 33.2 | 33.33333 | 0.139968 | 13.9968 | 3.333333333 | 0.111111111 |
| **FR03** | 1 | 4.4662 | 4.3717 | 4.5080 | 4.448633 | 30 | 33.2 | 33.8 | 33.9 | 33.63333 | 0.132268583 | 13.22685828 | 3.633333333 | 0.121111111 |
| **FR04** | 1 | 4.3018 | 4.4384 | 4.2843 | 4.3415 | 30 | 33.9 | 33.8 | 34.2 | 33.96667 | 0.127816487 | 12.78164868 | 3.966666667 | 0.132222222 |
| **FR05** | 1 | 4.2220 | 4.2094 | 4.1410 | 4.1908 | 30 | 32.9 | 33.8 | 33.5 | 33.4 | 0.125473054 | 12.54730539 | 3.4 | 0.113333333 |
| **FR06** | 1 | 4.5205 | 4.4228 | 4.3096 | 4.417633 | 30 | 33.9 | 33.7 | 34.8 | 34.13333 | 0.129422852 | 12.94228516 | 4.133333333 | 0.137777778 |
| **FR07** | 1 | 4.2310 | 4.3370 | 4.2686 | 4.278867 | 30 | 33.9 | 33.8 | 33.8 | 33.83333 | 0.126468966 | 12.64689655 | 3.833333333 | 0.127777778 |
| **FR10** | 1 | 4.8970 | 4.8321 | 4.8794 | 4.8695 | 30 | 33.9 | 34.1 | 33.8 | 33.93333 | 0.143501965 | 14.35019646 | 3.933333333 | 0.131111111 |
| **FR11** | 1 | 5.2086 | 5.3585 | 5.2125 | 5.259867 | 30 | 33.8 | 34.0 | 34.1 | 33.96667 | 0.154853778 | 15.48537782 | 3.966666667 | 0.132222222 |
| **FR12** | 1 | 5.3037 | 4.9768 | 4.8699 | 5.050133 | 30 | 34.2 | 34.8 | 34.7 | 34.56667 | 0.146098361 | 14.60983607 | 4.566666667 | 0.152222222 |
| **FR13** | 1 | 4.4013 | 4.4944 | 4.4781 | 4.457933 | 30 | 33.8 | 33.7 | 33.5 | 33.66667 | 0.132413861 | 13.24138614 | 3.666666667 | 0.122222222 |
| **FR14** | 1 | 4.8769 | 4.8567 | 4.8628 | 4.865467 | 30 | 33.9 | 34.0 | 34.0 | 33.96667 | 0.143242395 | 14.32423945 | 3.966666667 | 0.132222222 |
| **FR15** | 1 | 4.9209 | 4.7929 | 4.7967 | 4.836833 | 30 | 33.8 | 34.0 | 33.6 | 33.8 | 0.143101578 | 14.31015779 | 3.8 | 0.126666667 |
| **FR16** | 1 | 5.4546 | 5.3528 | 5.3662 | 5.3912 | 30 | 33.2 | 33.7 | 33.5 | 33.46667 | 0.161091633 | 16.10916335 | 3.466666667 | 0.115555556 |
| **FR17** | 1 | 5.2048 | 5.2809 | 5.1082 | 5.197967 | 30 | 34.1 | 34.0 | 34.0 | 34.03333 | 0.152731636 | 15.27316357 | 4.033333333 | 0.134444444 |
| **FR18** | 1 | 4.6264 | 4.5640 | 4.4659 | 4.5521 | 30 | 33.8 | 33.9 | 33.7 | 33.8 | 0.134677515 | 13.46775148 | 3.8 | 0.126666667 |
| **FR19** | 1 | 4.3801 | 4.3175 | 4.3776 | 4.3584 | 30 | 34.9 | 34.5 | 34.5 | 34.63333 | 0.125844081 | 12.58440808 | 4.633333333 | 0.154444444 |
| **US01** | 1 | 8.4462 | 8.8739 | 8.4292 | 8.5831 | 60 | 66.9 | 67.8 | 67.4 | 67.36667 | 0.127408709 | 12.74087086 | 7.366666667 | 0.122777778 |
| **US02** | 1 | 5.1030 | 5.0516 | 5.1426 | 5.099067 | 30 | 34.8 | 35.0 | 34.9 | 34.9 | 0.146105062 | 14.61050621 | 4.9 | 0.163333333 |
| **US03** | 1 | 8.7424 | 8.8899 | 8.8440 | 8.825433 | 60 | 66.1 | 65.9 | 66.1 | 66.03333 | 0.133651186 | 13.36511863 | 6.033333333 | 0.100555556 |
| **US04** | 1 | 8.6017 | 8.5708 | 8.3956 | 8.5227 | 60 | 66.2 | 66.2 | 68.4 | 66.93333 | 0.127331175 | 12.73311753 | 6.933333333 | 0.115555556 |
| **US05** | 1 | 8.7769 | 8.7224 | 8.6377 | 8.712333 | 60 | 65.8 | 67.0 | 65.8 | 66.2 | 0.131606244 | 13.16062437 | 6.2 | 0.103333333 |
| **US06** | 1 | 8.6877 | 8.7184 | 8.5705 | 8.658867 | 60 | 67.8 | 68.0 | 67.8 | 67.86667 | 0.127586444 | 12.7586444 | 7.866666667 | 0.131111111 |
| **US07** | 1 | 8.1659 | 8.0132 | 8.1068 | 8.0953 | 60 | 67.8 | 67.9 | 67.8 | 67.83333 | 0.119341032 | 11.93410319 | 7.833333333 | 0.130555556 |
| **US08** | 1 | 7.9151 | 7.7650 | 8.2540 | 7.978033 | 30 | 35.9 | 35.8 | 36.0 | 35.9 | 0.222229341 | 22.22293408 | 5.9 | 0.196666667 |
| **US09** | 1 | 4.1830 | 4.3330 | 4.6160 | 4.377333 | 30 | 33.7 | 33.8 | 33.6 | 33.7 | 0.129891197 | 12.98911968 | 3.7 | 0.123333333 |
| **US10** | 1 | 15.7764 | 15.6548 | 15.4805 | 15.63723 | 70 | 84.1 | 82.2 | 81.9 | 82.73333 | 0.189007655 | 18.90076551 | 12.73333333 | 0.181904762 |
| **US11** | 1 | 16.3577 | 17.3878 | 17.5891 | 17.11153 | 120 | 144.0 | 145.8 | 144.5 | 144.7667 | 0.118200783 | 11.82007829 | 24.76666667 | 0.206388889 |
| **US12** | 1 | 11.6445 | 11.5962 | 12.1916 | 11.81077 | 120 | 134.8 | 130.2 | 134.2 | 133.0667 | 0.088758267 | 8.875826653 | 13.06666667 | 0.108888889 |
| **US13** | 1 | 4.9512 | 5.0513 | 4.9483 | 4.9836 | 30 | 35.0 | 34.0 | 33.8 | 34.26667 | 0.145435798 | 14.54357977 | 4.266666667 | 0.142222222 |
| **US14** | 1 | 10.1176 | 10.3636 | 9.7932 | 10.09147 | 60 | 68.6 | 68.1 | 68.8 | 68.5 | 0.147320681 | 14.73206813 | 8.5 | 0.141666667 |
| **US15** | 1 | 9.1267 | 8.5719 | 8.5864 | 8.761667 | 60 | 68.2 | 68.1 | 68.4 | 68.23333 | 0.128407426 | 12.84074255 | 8.233333333 | 0.137222222 |
| **US16** | 1 | 8.5786 | 8.1567 | 8.5591 | 8.431467 | 60 | 67.9 | 68.0 | 68.0 | 67.96667 | 0.124052967 | 12.40529671 | 7.966666667 | 0.132777778 |
| **US17** | 1 | 10.1045 | 10.1541 | 10.0296 | 10.09607 | 60 | 69.4 | 69.7 | 70.0 | 69.7 | 0.144850311 | 14.48503109 | 9.7 | 0.161666667 |
| **US18** | 1 | 4.7271 | 4.2182 | 4.7081 | 4.551133 | 30 | 33.9 | 34.1 | 34.5 | 34.16667 | 0.133203902 | 13.32039024 | 4.166666667 | 0.138888889 |
| **US19** | 1 | 8.7122 | 8.5302 | 8.4398 | 8.560733 | 60 | 68.0 | 68.4 | 68.2 | 68.2 | 0.125523949 | 12.55239492 | 8.2 | 0.136666667 |
| **US20** | 1 | 9.7607 | 9.2714 | 9.4810 | 9.504367 | 60 | 68.2 | 68.2 | 68.0 | 68.13333 | 0.139496575 | 13.94965753 | 8.133333333 | 0.135555556 |
| **US21** | 1 | 8.5818 | 8.6508 | 8.7999 | 8.6775 | 60 | 68.1 | 68.0 | 68.5 | 68.2 | 0.12723607 | 12.72360704 | 8.2 | 0.136666667 |
| **US22** | 1 | 9.5458 | 10.0226 | 10.2440 | 9.937467 | 60 | 69.7 | 69.9 | 69.9 | 69.83333 | 0.142302625 | 14.23026253 | 9.833333333 | 0.163888889 |
| **US23** | 1 | 8.3789 | 8.7308 | 8.3795 | 8.4964 | 60 | 67.9 | 68.0 | 67.9 | 67.93333 | 0.125069676 | 12.50696762 | 7.933333333 | 0.132222222 |
| **US24** | 1 | 8.9021 | 8.4175 | 8.7682 | 8.695933 | 60 | 68.0 | 68.1 | 68.3 | 68.13333 | 0.127631115 | 12.76311155 | 8.133333333 | 0.135555556 |
| **US25** | 1 | 8.4933 | 8.2516 | 8.5421 | 8.429 | 60 | 67.8 | 68.1 | 68.3 | 68.06667 | 0.123834476 | 12.3834476 | 8.066666667 | 0.134444444 |
