# Supplementary material for: Manganese levels in infant formula and young child nutritional beverages in the United States and France: Comparison to breast milk and regulations
Source: PLoS One. 2019 Nov 5;14(11):e0223636. doi: 10.1371/journal.pone.0223636 (PMC6830775; doi:10.1371/journal.pone.0223636)
Supplement: S6 Table — (DOCX) [file pone.0223636.s006.docx]

**S6. Water and powder in 1 L of prepared formula**

| **Sample Number** | **Specified scoops / batch (scoops)** | **Specified water to add / batch (mL)** | **Average g powder / batch (g)** | **Average volume added by powder / batch (mL)** | **Average volume of prepared formula / batch (mL)** | **Average L of prepared / batch (L)** | **Batches / L (batches)** | **Water in 1 L of prepared formula (mL)** | **Powder in 1 L of prepared formula (g)** |
| --- | --- | --- | --- | --- | --- | --- | --- | --- | --- |
| **FR01** | 1 | 30 | 4.562867 | 3.166666667 | 33.16666667 | 0.033167 | 30.15075 | 904.5226 | 137.5739 |
| **FR02** | 1 | 30 | 4.6656 | 3.333333333 | 33.33333333 | 0.033333 | 30 | 900 | 139.968 |
| **FR03** | 1 | 30 | 4.448633 | 3.633333333 | 33.63333333 | 0.033633 | 29.73241 | 891.9722 | 132.2686 |
| **FR04** | 5 | 150 | 21.7075 | 19.83333333 | 169.8333333 | 0.169833 | 5.888126 | 883.2188 | 127.8165 |
| **FR05** | 3 | 90 | 12.5724 | 10.2 | 100.2 | 0.1002 | 9.98004 | 898.2036 | 125.4731 |
| **FR06** | 3 | 90 | 13.2529 | 12.4 | 102.4 | 0.1024 | 9.765625 | 878.9063 | 129.4229 |
| **FR07** | 3 | 90 | 12.8366 | 11.5 | 101.5 | 0.1015 | 9.852217 | 886.6995 | 126.469 |
| **FR10** | 3 | 90 | 14.6085 | 11.8 | 101.8 | 0.1018 | 9.823183 | 884.0864 | 143.502 |
| **FR11** | 7 | 210 | 36.81907 | 27.76666667 | 237.7666667 | 0.237767 | 4.205804 | 883.2188 | 154.8538 |
| **FR12** | 5 | 150 | 25.25067 | 22.83333333 | 172.8333333 | 0.172833 | 5.785921 | 867.8881 | 146.0984 |
| **FR13** | 6 | 180 | 26.7476 | 22 | 202 | 0.202 | 4.950495 | 891.0891 | 132.4139 |
| **FR14** | 1 | 30 | 4.865467 | 3.966666667 | 33.96666667 | 0.033967 | 29.44063 | 883.2188 | 143.2424 |
| **FR15** | 6 | 180 | 29.021 | 22.8 | 202.8 | 0.2028 | 4.930966 | 887.574 | 143.1016 |
| **FR16** | 1 | 30 | 5.3912 | 3.466666667 | 33.46666667 | 0.033467 | 29.88048 | 896.4143 | 161.0916 |
| **FR17** | 5 | 150 | 25.98983 | 20.16666667 | 170.1666667 | 0.170167 | 5.876592 | 881.4887 | 152.7316 |
| **FR18** | 5 | 150 | 22.7605 | 19 | 169 | 0.169 | 5.91716 | 887.574 | 134.6775 |
| **FR19** | 1 | 30 | 4.3584 | 4.633333333 | 34.63333333 | 0.034633 | 28.87392 | 866.2175 | 125.8441 |
| **US01** | 1 | 60 | 8.5831 | 7.366666667 | 67.36666667 | 0.067367 | 14.84414 | 890.6482 | 127.4087 |
| **US02** | 6 | 180 | 30.5944 | 29.4 | 209.4 | 0.2094 | 4.775549 | 859.5989 | 146.1051 |
| **US03** | 1 | 60 | 8.825433 | 6.033333333 | 66.03333333 | 0.066033 | 15.14387 | 908.632 | 133.6512 |
| **US04** | 1 | 60 | 8.5227 | 6.933333333 | 66.93333333 | 0.066933 | 14.94024 | 896.4143 | 127.3312 |
| **US05** | 1 | 60 | 8.712333 | 6.2 | 66.2 | 0.0662 | 15.10574 | 906.3444 | 131.6062 |
| **US06** | 1 | 60 | 8.658867 | 7.866666667 | 67.86666667 | 0.067867 | 14.73477 | 884.0864 | 127.5864 |
| **US07** | 1 | 60 | 8.0953 | 7.833333333 | 67.83333333 | 0.067833 | 14.74201 | 884.5209 | 119.341 |
| **US08** | 4 | 120 | 31.91213 | 23.6 | 143.6 | 0.1436 | 6.963788 | 835.6546 | 222.2293 |
| **US09** | 1 | 30 | 4.377333 | 3.7 | 33.7 | 0.0337 | 29.67359 | 890.2077 | 129.8912 |
| **US10** | 3 | 210 | 46.9117 | 38.2 | 248.2 | 0.2482 | 4.029009 | 846.0919 | 189.0077 |
| **US11** | 2 | 240 | 34.22307 | 49.53333333 | 289.5333333 | 0.289533 | 3.453834 | 828.9201 | 118.2008 |
| **US12** | 2 | 240 | 23.62153 | 26.13333333 | 266.1333333 | 0.266133 | 3.757515 | 901.8036 | 88.75827 |
| **US13** | 2 | 60 | 9.9672 | 8.533333333 | 68.53333333 | 0.068533 | 14.59144 | 875.4864 | 145.4358 |
| **US14** | 1 | 60 | 10.09147 | 8.5 | 68.5 | 0.0685 | 14.59854 | 875.9124 | 147.3207 |
| **US15** | 1 | 60 | 8.761667 | 8.233333333 | 68.23333333 | 0.068233 | 14.65559 | 879.3356 | 128.4074 |
| **US16** | 1 | 60 | 8.431467 | 7.966666667 | 67.96666667 | 0.067967 | 14.71309 | 882.7857 | 124.053 |
| **US17** | 1 | 60 | 10.09607 | 9.7 | 69.7 | 0.0697 | 14.3472 | 860.8321 | 144.8503 |
| **US18** | 2 | 60 | 9.102267 | 8.333333333 | 68.33333333 | 0.068333 | 14.63415 | 878.0488 | 133.2039 |
| **US19** | 1 | 60 | 8.560733 | 8.2 | 68.2 | 0.0682 | 14.66276 | 879.7654 | 125.5239 |
| **US20** | 1 | 60 | 9.504367 | 8.133333333 | 68.13333333 | 0.068133 | 14.6771 | 880.6262 | 139.4966 |
| **US21** | 1 | 60 | 8.6775 | 8.2 | 68.2 | 0.0682 | 14.66276 | 879.7654 | 127.2361 |
| **US22** | 1 | 60 | 9.937467 | 9.833333333 | 69.83333333 | 0.069833 | 14.31981 | 859.1885 | 142.3026 |
| **US23** | 1 | 60 | 8.4964 | 7.933333333 | 67.93333333 | 0.067933 | 14.72031 | 883.2188 | 125.0697 |
| **US24** | 1 | 60 | 8.695933 | 8.133333333 | 68.13333333 | 0.068133 | 14.6771 | 880.6262 | 127.6311 |
| **US25** | 1 | 60 | 8.429 | 8.066666667 | 68.06666667 | 0.068067 | 14.69148 | 881.4887 | 123.8345 |
