# Supplementary material for: Manganese levels in infant formula and young child nutritional beverages in the United States and France: Comparison to breast milk and regulations
Source: PLoS One. 2019 Nov 5;14(11):e0223636. doi: 10.1371/journal.pone.0223636 (PMC6830775; doi:10.1371/journal.pone.0223636)
Supplement: S7 Table — (DOCX) [file pone.0223636.s007.docx]

**S7. Mass of solids in 1 L of liquid samples (by evaporation starting with measured volumes)**

| **Sample Number** | **g solids / 10 mL liquid product--Rep 1 (g)** | **g solids / 10 mL liquid product--Rep 2 (g)** | **g solids / 10 mL liquid product--Rep 3 (g)** | **Average g solids / 10 mL liquid product (g)** | **Average solids / 1 mL liquid product (g)** | **Average g solids / 1 L liquid product (g)** |
| --- | --- | --- | --- | --- | --- | --- |
| **FR08** | 2.0590 | 2.0193 | 2.0674 | 2.0486 | 0.204857 | 204.8567 |
| **FR09** | 1.9444 | 1.8631 | 1.8742 | 1.8939 | 0.18939 | 189.39 |
