# Supplementary material for: Manganese levels in infant formula and young child nutritional beverages in the United States and France: Comparison to breast milk and regulations
Source: PLoS One. 2019 Nov 5;14(11):e0223636. doi: 10.1371/journal.pone.0223636 (PMC6830775; doi:10.1371/journal.pone.0223636)
Supplement: S8 Table — (DOCX) [file pone.0223636.s008.docx]

**S8. Calculations to derive g powder (or solids) / 100 kCal based on laboratory measurements of g powder (or solids) / L prepared formula and labeled energy content**

*By US labeling law, 1 "Calorie" = 1 kCal*

*By US labeling law, 1 oz = 30 mL (21 CFR 101.9, 2017)*

| **Color key** |  | *=as stated on label* |
| --- | --- | --- |

| **Sample Number** | **"Calories" / Serving (kCal)** | **Serving size (oz)** | **Serving size (mL)** | **Serving size (g powder)** | **kCal / 100 g powder (kCal)** | **kCal / 100 mL prepared formula** | **Serving size (mL)** | **kCal / mL (kCal)** | **kCal / 100 mL (kCal)** | **g / mL prepared formula (g)** | **g / 100 mL prepared formula (g)** | **kCal / 1 g solids (kCal)** | **g solids / 1 kCal (g)** | **g solids / 100 kCal (g)** |
| --- | --- | --- | --- | --- | --- | --- | --- | --- | --- | --- | --- | --- | --- | --- |
| **FR01** |  |  |  |  | 484 |  |  |  |  |  |  | 4.84 | 0.206612 | 20.66116 |
| **FR02** |  |  |  |  | 451 |  |  |  |  |  |  | 4.51 | 0.221729 | 22.17295 |
| **FR03** |  |  |  |  | 467 |  |  |  |  |  |  | 4.67 | 0.214133 | 21.41328 |
| **FR04** |  |  |  |  | 496 |  |  |  |  |  |  | 4.96 | 0.201613 | 20.16129 |
| **FR05** |  |  |  |  | 506 |  |  |  |  |  |  | 5.06 | 0.197628 | 19.76285 |
| **FR06** |  |  |  |  | 515 |  |  |  |  |  |  | 5.15 | 0.194175 | 19.41748 |
| **FR07** |  |  |  |  | 517 |  |  |  |  |  |  | 5.17 | 0.193424 | 19.34236 |
| **FR08** |  |  |  |  |  | 86 |  |  |  |  |  | 4.198057 | 0.238205 | 23.82054 |
| **FR09** |  |  |  |  |  | 84 |  |  |  |  |  | 4.435292 | 0.225464 | 22.54643 |
| **FR10** |  |  |  |  |  | 67 |  |  |  |  | 14.3502 |  | 0.214182 | 21.4182 |
| **FR11** |  |  |  |  | 483 |  |  |  |  |  |  | 4.83 | 0.207039 | 20.70393 |
| **FR12** |  |  |  |  | 484 |  |  |  |  |  |  | 4.84 | 0.206612 | 20.66116 |
| **FR13** |  |  |  |  |  | 64 |  |  |  |  | 13.24139 |  | 0.206897 | 20.68967 |
| **FR14** |  |  |  |  | 480 |  |  |  |  |  |  | 4.8 | 0.208333 | 20.83333 |
| **FR15** |  |  |  |  |  | 70 |  |  |  |  | 14.31016 |  | 0.204431 | 20.44308 |
| **FR16** |  |  |  |  |  | 68 |  |  |  |  | 16.10916 |  | 0.236899 | 23.68995 |
| **FR17** |  |  |  |  |  | 66 |  |  |  |  | 15.27316 |  | 0.231412 | 23.14116 |
| **FR18** |  |  |  |  | 481 |  |  |  |  |  |  | 4.81 | 0.2079 | 20.79002 |
| **FR19** |  |  |  |  | 477.4 |  |  |  |  |  |  | 4.774 | 0.209468 | 20.9468 |
| **US01** | 100 | 5.3 |  |  |  |  | 159 | 0.628930818 | 62.89308 | 0.127409 | 12.74087 |  |  | 20.25798 |
| **US02** |  |  |  |  | 496.5 |  |  |  |  |  |  | 4.965 | 0.20141 | 20.14099 |
| **US03** | 100 | 5 |  |  |  |  | 150 | 0.666666667 | 66.66667 | 0.133651 | 13.36512 |  |  | 20.04768 |
| **US04** | 100 | 5 |  | 21 |  |  |  |  |  |  |  | 4.761905 | 0.21 | 21 |
| **US05** | 100 | 5 |  | 21 |  |  |  |  |  |  |  | 4.761905 | 0.21 | 21 |
| **US06** | 100 | 5 |  |  |  |  | 150 | 0.666666667 | 66.66667 | 0.127586 | 12.75864 |  |  | 19.13797 |
| **US07** | 100 | 5 | 150 |  |  |  | 150 | 0.666666667 | 66.66667 | 0.119341 | 11.9341 |  |  | 17.90115 |
| **US08** | 100 |  | 100 |  | 451 |  |  |  |  |  |  | 4.51 | 0.221729 | 22.17295 |
| **US09** |  | 6 | 180 | 27 |  | 67 |  |  |  | 0.129891 | 12.98912 |  | 0.193867 | 19.38675 |
| **US10** | 240 | 8 | 240 | 52 |  |  |  |  |  |  |  | 4.615385 | 0.216667 | 21.66667 |
| **US11** | 170 | 8 |  | 40 |  |  |  |  |  |  |  | 4.25 | 0.235294 | 23.52941 |
| **US12** | 100 | 8 |  | 27 |  |  |  |  |  |  |  | 3.703704 | 0.27 | 27 |
| **US13** | 90 | 4 | 120 | 19 |  |  |  |  |  |  |  | 4.736842 | 0.211111 | 21.11111 |
| **US14** | 100 | 5 |  |  | 475 |  |  |  |  |  |  | 4.75 | 0.210526 | 21.05263 |
| **US15** | 100 | 5 |  |  |  |  | 150 | 0.666666667 | 66.66667 | 0.128407 | 12.84074 |  |  | 19.26111 |
| **US16** | 100 | 5 |  |  |  |  | 150 | 0.666666667 | 66.66667 | 0.124053 | 12.4053 |  |  | 18.60795 |
| **US17** | 100 | 5 |  |  |  |  | 150 | 0.666666667 | 66.66667 | 0.14485 | 14.48503 |  |  | 21.72755 |
| **US18** | 100 | 5 |  |  |  |  | 150 | 0.666666667 | 66.66667 | 0.133204 | 13.32039 |  |  | 19.98059 |
| **US19** | 100 | 5 | 150 |  |  |  | 150 | 0.666666667 | 66.66667 | 0.125524 | 12.55239 |  |  | 18.82859 |
| **US20** | 100 | 5 |  |  |  |  | 150 | 0.666666667 | 66.66667 | 0.139497 | 13.94966 |  |  | 20.92449 |
| **US21** | 100 | 5 |  |  |  |  | 150 | 0.666666667 | 66.66667 | 0.127236 | 12.72361 |  |  | 19.08541 |
| **US22** | 100 | 5 |  |  |  |  | 150 | 0.666666667 | 66.66667 | 0.142303 | 14.23026 |  |  | 21.34539 |
| **US23** | 100 | 5 | 150 |  |  |  | 150 | 0.666666667 | 66.66667 | 0.12507 | 12.50697 |  |  | 18.76045 |
| **US24** | 100 | 5 | 150 |  |  |  | 150 | 0.666666667 | 66.66667 | 0.127631 | 12.76311 |  |  | 19.14467 |
| **US25** | 100 | 5 |  |  |  |  | 150 | 0.666666667 | 66.66667 | 0.123834 | 12.38345 |  |  | 18.57517 |
