# Supplementary material for: Manganese levels in infant formula and young child nutritional beverages in the United States and France: Comparison to breast milk and regulations
Source: PLoS One. 2019 Nov 5;14(11):e0223636. doi: 10.1371/journal.pone.0223636 (PMC6830775; doi:10.1371/journal.pone.0223636)
Supplement: S9 Table — (DOCX) [file pone.0223636.s009.docx]

**S9. PIXE Data**

| \| **Sample Number** \| **Mn (µg/g)** \| **Mean Mn (µg/g)** \| ***s*** \| \| --- \| --- \| --- \| --- \| \| FR01 \|  \| 1.713 \| 0.43417 \| \| FR01a \| 1.543 \|  \|  \| \| FR01b \| 1.389 \|  \|  \| \| FR01c \| 2.206 \|  \|  \| \| FR02 \|  \| 2.089 \| 0.60778 \| \| FR02a \| 1.400 \|  \|  \| \| FR02b \| 2.548 \|  \|  \| \| FR02c \| 2.319 \|  \|  \| \| FR03 \|  \| 1.714 \| 0.24041 \| \| FR03a \| 1.530 \|  \|  \| \| FR03b \| 1.986 \|  \|  \| \| FR03c \| 1.627 \|  \|  \| \| FR04 \|  \| 2.262 \| 0.58188 \| \| FR04a \| 1.592 \|  \|  \| \| FR04b \| 2.548 \|  \|  \| \| FR04c \| 2.645 \|  \|  \| \| FR05 \|  \| 1.734 \| 0.70127 \| \| FR05a \| 1.210 \|  \|  \| \| FR05b \| 1.463 \|  \|  \| \| FR05c \| 2.531 \|  \|  \| \| FR06 \|  \| 2.378 \| 0.17814 \| \| FR06a \| 2.570 \|  \|  \| \| FR06b \| 2.346 \|  \|  \| \| FR06c \| 2.218 \|  \|  \| \| FR07 \|  \| 2.738 \| 0.35536 \| \| FR07a \| 2.571 \|  \|  \| \| FR07b \| 3.146 \|  \|  \| \| FR07c \| 2.497 \|  \|  \| \| FR08 \|  \| 5.860 \| 0.77667 \| \| FR08a \| 5.035 \|  \|  \| \| FR08b \| 5.967 \|  \|  \| \| FR08c \| 6.577 \|  \|  \| \| FR09 \|  \| 4.565 \| 0.54468 \| \| FR09a \| 5.094 \|  \|  \| \| FR09b \| 4.595 \|  \|  \| \| FR09c \| 4.005 \|  \|  \| \| FR10 \|  \| 2.454 \| 0.65133 \| \| FR10a \| 3.206 \|  \|  \| \| FR10b \| 2.081 \|  \|  \| \| FR10c \| 2.075 \|  \|  \| \| FR11 \|  \| 2.041 \| 0.60427 \| \| FR11a \| 1.504 \|  \|  \| \| FR11b \| 2.695 \|  \|  \| \| FR11c \| 1.924 \|  \|  \| \| FR12 \|  \| 2.644 \| 0.86049 \| \| FR12a \| 2.145 \|  \|  \| \| FR12b \| 3.638 \|  \|  \| \| FR12c \| 2.150 \|  \|  \| \| FR13 \|  \| 1.539 \| 0.60688 \| \| FR13a \| 1.052 \|  \|  \| \| FR13b \| 2.219 \|  \|  \| \| FR13c \| 1.346 \|  \|  \| \| FR14 \|  \| 2.094 \| 0.30087 \| \| FR14a \| 2.319 \|  \|  \| \| FR14b \| 2.210 \|  \|  \| \| FR14c \| 1.752 \|  \|  \| \| FR15 \|  \| 1.956 \| 0.43559 \| \| FR15a \| 2.395 \|  \|  \| \| FR15b \| 1.524 \|  \|  \| \| FR15c \| 1.949 \|  \|  \| \| FR16 \|  \| 1.982 \| 0.50203 \| \| FR16a \| 2.252 \|  \|  \| \| FR16b \| 2.291 \|  \|  \| \| FR16c \| 1.403 \|  \|  \| \| FR17 \|  \| 1.888 \| 0.29821 \| \| FR17a \| 1.577 \|  \|  \| \| FR17b \| 2.172 \|  \|  \| \| FR17c \| 1.913 \|  \|  \| \| FR18 \|  \| 2.515 \| 0.2248 \| \| FR18a \| 2.520 \|  \|  \| \| FR18b \| 2.288 \|  \|  \| \| FR18c \| 2.737 \|  \|  \| \| FR19 \|  \| 4.422 \| 0.1658 \| \| FR19a \| 4.503 \|  \|  \| \| FR19b \| 4.533 \|  \|  \| \| FR19c \| 4.232 \|  \|  \| \| US01 \|  \| 1.780 \| 0.71453 \| \| US01a \| 2.546 \|  \|  \| \| US01b \| 1.132 \|  \|  \| \| US01c \| 1.661 \|  \|  \| \| US02 \|  \| 2.716 \| 0.26823 \| \| US02a \| 2.661 \|  \|  \| \| US02b \| 2.479 \|  \|  \| \| US02c \| 3.007 \|  \|  \| \| US03 \|  \| 2.332 \| 0.53247 \| \| US03a \| 2.202 \|  \|  \| \| US03b \| 2.918 \|  \|  \| \| US03c \| 1.877 \|  \|  \| \| US04 \|  \| 1.262 \| 0.18129 \| \| US04a \| 1.420 \|  \|  \| \| US04b \| 1.064 \|  \|  \| \| US04c \| 1.300 \|  \|  \| \| US05 \|  \| 7.881 \| 0.64888 \| \| US05a \| 8.405 \|  \|  \| \| US05b \| 7.155 \|  \|  \| \| US05c \| 8.083 \|  \|  \| \| US06 \|  \| 3.376 \| 0.63817 \| \| US06a \| 2.876 \|  \|  \| \| US06b \| 4.095 \|  \|  \| \| US06c \| 3.158 \|  \|  \| \| US07 \|  \| 2.694 \| 0.57921 \| \| US07a \| 2.618 \|  \|  \| \| US07b \| 2.157 \|  \|  \| \| US07c \| 3.308 \|  \|  \| \| US08 \|  \| 7.357 \| 0.43672 \| \| US08a \| 6.853 \|  \|  \| \| US08b \| 7.597 \|  \|  \| \| US08c \| 7.622 \|  \|  \| \| US09 \|  \| 2.604 \| 0.51942 \| \| US09a \| 2.314 \|  \|  \| \| US09b \| 3.203 \|  \|  \| \| US09c \| 2.293 \|  \|  \| \| US10 \|  \| 11.197 \| 1.83651 \| \| US10a \| 12.560 \|  \|  \| \| US10b \| 11.921 \|  \|  \| \| US10c \| 9.109 \|  \|  \| \| US11 \|  \| 9.216 \| 0.83906 \| \| US11a \| 9.969 \|  \|  \| \| US11b \| 9.368 \|  \|  \| \| US11c \| 8.311 \|  \|  \| \| US12 \|  \| 31.847 \| 2.74768 \| \| US12a \| 33.934 \|  \|  \| \| US12b \| 32.874 \|  \|  \| \| US12c \| 28.734 \|  \|  \| \| US13 \|  \| 2.243 \| 0.33493 \| \| US13a \| 2.615 \|  \|  \| \| US13b \| 2.148 \|  \|  \| \| US13c \| 1.966 \|  \|  \| \| US14 \|  \| 5.656 \| 0.54088 \| \| US14a \| 5.915 \|  \|  \| \| US14b \| 6.018 \|  \|  \| \| US14c \| 5.034 \|  \|  \| \| US15 \|  \| 4.226 \| 0.67649 \| \| US15a \| 5.004 \|  \|  \| \| US15b \| 3.898 \|  \|  \| \| US15c \| 3.777 \|  \|  \| \| US16 \|  \| 3.876 \| 0.77499 \| \| US16a \| 3.269 \|  \|  \| \| US16b \| 3.611 \|  \|  \| \| US16c \| 4.749 \|  \|  \| \| US17 \|  \| 2.930 \| 0.09888 \| \| US17a \| 2.980 \|  \|  \| \| US17b \| 2.994 \|  \|  \| \| US17c \| 2.816 \|  \|  \| \| US18 \|  \| 1.563 \| 0.65391 \| \| US18a \| 1.067 \|  \|  \| \| US18b \| 2.304 \|  \|  \| \| US18c \| 1.318 \|  \|  \| \| US19 \|  \| 6.324 \| 0.39723 \| \| US19a \| 6.437 \|  \|  \| \| US19b \| 6.653 \|  \|  \| \| US19c \| 5.883 \|  \|  \| \| US20 \|  \| 1.641 \| 0.30776 \| \| US20a \| 1.597 \|  \|  \| \| US20b \| 1.357 \|  \|  \| \| US20c \| 1.968 \|  \|  \| \| US21 \|  \| 2.691 \| 0.61247 \| \| US21a \| 2.051 \|  \|  \| \| US21b \| 3.271 \|  \|  \| \| US21c \| 2.750 \|  \|  \| \| US22 \|  \| 2.948 \| 0.08111 \| \| US22a \| 2.954 \|  \|  \| \| US22b \| 3.027 \|  \|  \| \| US22c \| 2.865 \|  \|  \| \| US23 \|  \| 2.074 \| 0.29428 \| \| US23a \| 2.409 \|  \|  \| \| US23b \| 1.957 \|  \|  \| \| US23c \| 1.857 \|  \|  \| \| US24 \|  \| 2.505 \| 0.66655 \| \| US24a \| 1.852 \|  \|  \| \| US24b \| 3.184 \|  \|  \| \| US24c \| 2.478 \|  \|  \| \| US25 \|  \| 2.076 \| 0.85932 \| \| US25a \| 1.149 \|  \|  \| \| US25b \| 2.845 \|  \|  \| \| US25c \| 2.235 \|  \|  \| |  |  |  |
| --- | --- | --- | --- | --- | --- | --- | --- | --- | --- | --- | --- | --- | --- | --- | --- | --- | --- | --- | --- | --- | --- | --- | --- | --- | --- | --- | --- | --- | --- | --- | --- | --- | --- | --- | --- | --- | --- | --- | --- | --- | --- | --- | --- | --- | --- | --- | --- | --- | --- | --- | --- | --- | --- | --- | --- | --- | --- | --- | --- | --- | --- | --- | --- | --- | --- | --- | --- | --- | --- | --- | --- | --- | --- | --- | --- | --- | --- | --- | --- | --- | --- | --- | --- | --- | --- | --- | --- | --- | --- | --- | --- | --- | --- | --- | --- | --- | --- | --- | --- | --- | --- | --- | --- | --- | --- | --- | --- | --- | --- | --- | --- | --- | --- | --- | --- | --- | --- | --- | --- | --- | --- | --- | --- | --- | --- | --- | --- | --- | --- | --- | --- | --- | --- | --- | --- | --- | --- | --- | --- | --- | --- | --- | --- | --- | --- | --- | --- | --- | --- | --- | --- | --- | --- | --- | --- | --- | --- | --- | --- | --- | --- | --- | --- | --- | --- | --- | --- | --- | --- | --- | --- | --- | --- | --- | --- | --- | --- | --- | --- | --- | --- | --- | --- | --- | --- | --- | --- | --- | --- | --- | --- | --- | --- | --- | --- | --- | --- | --- | --- | --- | --- | --- | --- | --- | --- | --- | --- | --- | --- | --- | --- | --- | --- | --- | --- | --- | --- | --- | --- | --- | --- | --- | --- | --- | --- | --- | --- | --- | --- | --- | --- | --- | --- | --- | --- | --- | --- | --- | --- | --- | --- | --- | --- | --- | --- | --- | --- | --- | --- | --- | --- | --- | --- | --- | --- | --- | --- | --- | --- | --- | --- | --- | --- | --- | --- | --- | --- | --- | --- | --- | --- | --- | --- | --- | --- | --- | --- | --- | --- | --- | --- | --- | --- | --- | --- | --- | --- | --- | --- | --- | --- | --- | --- | --- | --- | --- | --- | --- | --- | --- | --- | --- | --- | --- | --- | --- | --- | --- | --- | --- | --- | --- | --- | --- | --- | --- | --- | --- | --- | --- | --- | --- | --- | --- | --- | --- | --- | --- | --- | --- | --- | --- | --- | --- | --- | --- | --- | --- | --- | --- | --- | --- | --- | --- | --- | --- | --- | --- | --- | --- | --- | --- | --- | --- | --- | --- | --- | --- | --- | --- | --- | --- | --- | --- | --- | --- | --- | --- | --- | --- | --- | --- | --- | --- | --- | --- | --- | --- | --- | --- | --- | --- | --- | --- | --- | --- | --- | --- | --- | --- | --- | --- | --- | --- | --- | --- | --- | --- | --- | --- | --- | --- | --- | --- | --- | --- | --- | --- | --- | --- | --- | --- | --- | --- | --- | --- | --- | --- | --- | --- | --- | --- | --- | --- | --- | --- | --- | --- | --- | --- | --- | --- | --- | --- | --- | --- | --- | --- | --- | --- | --- | --- | --- | --- | --- | --- | --- | --- | --- | --- | --- | --- | --- | --- | --- | --- | --- | --- | --- | --- | --- | --- | --- | --- | --- | --- | --- | --- | --- | --- | --- | --- | --- | --- | --- | --- | --- | --- | --- | --- | --- | --- | --- | --- | --- | --- | --- | --- | --- | --- | --- | --- | --- | --- | --- | --- | --- | --- | --- | --- | --- | --- | --- | --- | --- | --- | --- | --- | --- | --- | --- | --- | --- | --- | --- | --- | --- | --- | --- | --- | --- | --- | --- | --- | --- | --- | --- | --- | --- | --- | --- | --- | --- | --- | --- | --- | --- | --- | --- | --- | --- | --- | --- | --- | --- | --- | --- | --- | --- | --- | --- | --- | --- | --- | --- | --- | --- | --- | --- | --- | --- | --- | --- | --- | --- | --- | --- | --- | --- | --- | --- | --- | --- | --- | --- | --- | --- | --- | --- | --- | --- | --- | --- | --- | --- | --- | --- | --- | --- | --- | --- | --- | --- | --- | --- | --- | --- | --- | --- | --- | --- | --- | --- | --- | --- | --- | --- | --- | --- | --- | --- | --- | --- | --- | --- | --- | --- | --- | --- | --- | --- | --- | --- | --- | --- | --- | --- | --- | --- | --- | --- | --- | --- | --- | --- | --- | --- | --- | --- | --- | --- | --- | --- | --- | --- | --- | --- | --- | --- | --- | --- | --- | --- | --- | --- | --- | --- | --- | --- | --- | --- | --- | --- | --- | --- | --- | --- | --- | --- | --- | --- | --- | --- | --- | --- | --- | --- | --- | --- | --- | --- | --- | --- | --- | --- | --- | --- | --- | --- | --- | --- | --- | --- | --- | --- | --- | --- | --- | --- | --- | --- | --- | --- | --- | --- | --- | --- | --- | --- | --- | --- |
