# Supplementary material for: Manganese levels in infant formula and young child nutritional beverages in the United States and France: Comparison to breast milk and regulations
Source: PLoS One. 2019 Nov 5;14(11):e0223636. doi: 10.1371/journal.pone.0223636 (PMC6830775; doi:10.1371/journal.pone.0223636)
Supplement: S10 Table — (DOCX) [file pone.0223636.s010.docx]

**S.10 Mean NIST measurements**

|  | |
| --- | --- |
|  | **Mn (µg/g)** |
| **NIST1** | 50.40 |
| **NIST2** | 47.62 |
| **NIST3** | 50.42 |
| **mean** | 49.48 |
|  |  |
| **NIST 1849A certified values** | |
|  | **Mn (µg/g)** |
|  | 49.59 |
|  |  |
| **% Relative error** | |
|  | -0.22 |
